# Impact of Genetic Variants Associated with Neurodevelopmental Disorders on the WAVE Regulatory Complex

**DOI:** 10.1021/acs.jcim.5c01162

**Published:** 2025-07-09

**Authors:** Song Xie, Ke Zuo, Silvia De Rubeis, Giorgio Bonollo, Giorgio Colombo, Paolo Ruggerone, Paolo Carloni

**Affiliations:** † Computational Biomedicine, Institute of Neuroscience and Medicine INM-9, Forschungszentrum Jülich GmbH, 52428 Jülich, Germany; ‡ Department of Physics, RWTH Aachen University, 52056 Aachen, Germany; § National & Local Joint Engineering Research Center of Targeted and Innovative Therapeutics, Chongqing Key Laboratory of Kinase Modulators as Innovative Medicine, College of Pharmacy (International Academy of Targeted Therapeutics and Innovation), Chongqing University of Arts and Sciences, 5414556 Chongqing, China; ∥ Department of Physics, University of Cagliari, Monserrato, 09042 Cagliari, Italy; ⊥ Seaver Autism Center for Research and Treatment, Icahn School of Medicine at Mount Sinai, New York 10029, United States; # Department of Psychiatry, Icahn School of Medicine at Mount Sinai, New York 10029, United States; ∇ The Mindich Child Health and Development Institute, Icahn School of Medicine at Mount Sinai, New York 10029-5674, United States; ⬡ Friedman Brain Institute, Icahn School of Medicine at Mount Sinai, New York 10029-6574, United States; ◆ Department of Pharmacological Sciences, Icahn School of Medicine at Mount Sinai, New York 10029-5674, United States; ¶ Alper Center for Neural Development and Regeneration Friedman Brain Institute, Icahn School of Medicine at Mount Sinai, New York 10029-5674, United States; ■ Dipartimento di Chimica, Università di Pavia, Via Taramelli 12, 27100 Pavia, Italy; ● JARA Institute: Molecular Neuroscience and Imaging, Institute of Neuroscience and Medicine INM-11, Forschungszentrum Jülich GmbH, 09042 Jülich, Germany

## Abstract

The WAVE regulatory complex (WRC) is a heteropentamer necessary for the regulation of actin cytoskeleton. Genetic variants in components of the WRC have been associated with increased risk for neurodevelopmental disorders (NDDs), including autism spectrum disorder (ASD). Some of the missense variants associated with NDDs have been reported to cause aberrant detachment of the WRC domain active C-terminal region (ACR). Using molecular dynamics simulations, we show that these variants cause a similar increase in disorder in a specific helix of ACR as well as a decrease in ACR/WRC interactions, irrespective of their position and chemical nature. These might assist ACR detachment. We further demonstrate their impact on the large-scale motions of the complex. Taken together, these results do suggest that different modifications associated with the NDD-associated variants converge on similar changes to the structural dynamics of the WRC.

## Graphical abstract

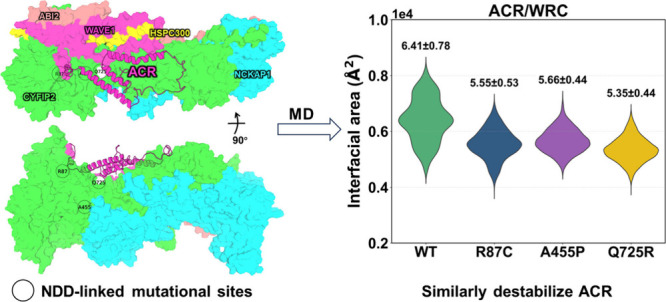

## 1 Introduction

Neurodevelopmental disorders (NDDs) make up a group of conditions typically emerging during childhood and resulting from alterations in brain development. Autism spectrum disorder (ASD) is a clinically heterogeneous NDD characterized by deficits in social communication and interaction, as well as repetitive behavior/restricted interests.^1,2^ ASD affects about 1% of individuals worldwide^3^ and often requires lifelong support.^4^ NDDs, including ASD, have a strong genetic component.^5–7^ Hundreds of risk genes for ASD have been identified by large-scale sequencing studies.^5^ Among them are genes encoding for regulators of the cytoskeleton,^8–11^ including *CYFIP2* (cytoplasmic FMR1-Interacting protein 2) and *NCKAP1* (non-Catalytic region of tyrosine kinase associated protein 1). These two genes encode core components of the WAVE regulatory complex (WRC), a highly conserved heteropentameric complex that regulates actin remodelling. The WRC comprises an elongated pseudosymmetric dimer formed by CYFIP1/2 and NCKAP1 and then a trimer consisting of ABI1/2/3, HSPC300, and WAVE1/2/3.^12,13^ WRC activation triggers actin remodeling^14^ (Figure 1 and Table S1), and this process is essential for brain development and function, including synapse formation and maturation.^15,16^ The activation requires the detachment of the active C-terminal region (ACR) from the rest of WRC (Figure 1).^17^ In physiological processes, this is caused by the binding of cellular partners (such as the GTPase Rac1 ^18^) to the complex (Figure S1).^17^ Notably, the conversion from the initial, “inactive” conformation of WRC to the “active” one (in which ACR is detached) has been show to occur without binding events when CYFIP2 carries missense mutations associated with NDDs (e.g., A455P, R87C, I664M, E665K, D724H, and Q725R) (Figure 1 and Table S1). [Other 12 variants in CYFIP2 (Tab S1) are associated with NDDs.^26^ The Y108H variant aberrantly enhances the binding of Rac1,^19^ while the effect of the others is not known.][Bibr ref19] This aberrant activation of the WRC may disrupt spine morphology, excitatory/inhibitory balance, and/or neuronal excitability,^15,20^ ultimately leading to ASD and other NDDs.^9,21^ The pathological nature of mutations is investigated by sequence-based methods, including PMUT^22^ and MutPred2 ^23^ servers. Both servers classify the mutations as pathogenic (pathogenic scores >0.5; Table S2).

**Figure 1:**
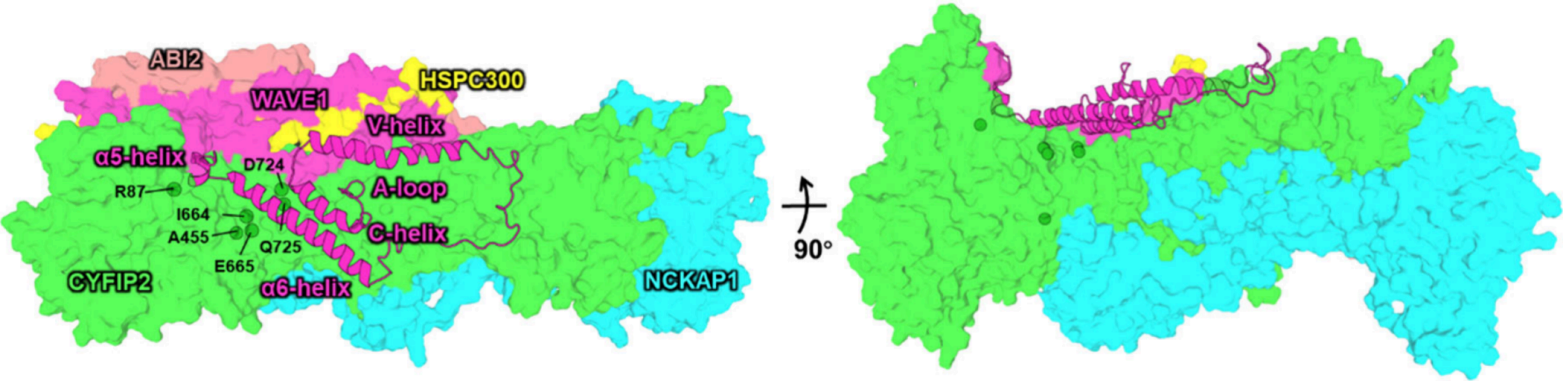
Schematic of WRC inactive form. The WRC consists of CYFIP2 (green), NCKAP1 (cyan), WAVE1 (magenta), HSPC300 (yellow), and ABI2 (peach). Subunits are displayed as surface representations, except for ACR (cartoon). The latter involves the α5 and α6 helices, the functional region (A loop and V and C helices), and loop links. The mutation sites discussed in the text are labeled. The model shown is a representative MD structure, which is based on Alphafold3 ^25^ and an X-ray study.^12^

Cryo-EM and X-ray studies have provided key insights into the two conformations of the WCR.^12,18^ In the inactive WCR, the ACR has a well-defined secondary structure (four extended helices connected by loops, see Figure 1).^12,18^ In the active WRC, the ACR separates from the rest of the complex and its helices unfold to an intrinsically disordered region (Figure 1 and Figure S1), whose structural determinants are not known.^8^ Some of the motifs in the ACR, including the A-loop, V- and C-helices (VCA), can then bind and activate the Arp2/3 complex, leading to actin remodelling.^24^ The hallmark of this process is the formation of lamellipodia.^13^

How the six CYFIP2 NDD-linked missense variants affect both states is not fully known. One of them (A455P) is located internally to the protein, while the other five are located at the ACR/CYFIP2 interface.^26^ Our earlier docking-based study suggests that such variants alter the interactions at the interfaces relative to the complex.^26^ Several crucial questions remain yet unanswered for all of these mutations, including how they produce a similar effect, i.e., altering the functional state and activity of WRC by propagation of structural and dynamic changes through the complex via allosteric pathways^19,26^ and/or by modifying directly structural determinants.^19^

To gain insights into WRC aberrant activation, we perform molecular dynamics (MD) simulations on three CYFIP2 mutants of the inactive form (A455P, R87C, and Q725R, Figure 1 and Table S1) and compare them with MD simulations of the wild-type (WT) complex. Specifically, for each system we perform three independent 2-μs all-atom replicas (Table S3–S5) and assess the impact on local chemical environments, the stability of subunit–subunit interfaces, large-scale motions, and structural dynamics of the ACR.

## 2 Results and Discussion

For each system, 4.5-μs-long equilibrated trajectories are pooled from the three 2-μs-long replicas to perform analysis (Figures S2–S4). No subunit dissociation or significant unfolding is observed in all systems (Figure S5).

### Positions Undergoing Mutations

In the WT WRC (Figure 2A), (i) R87­(CYFIP2) establishes an H-bond with Y151­(ACR) with a high occupancy (84%; Table S6). (ii) A455­(CYFIP2) (Figure 2B and Table S6) forms van der Waals interactions with Y687 and Y690 (both CYFIP2). (iii) Q725­(CYFIP2) forms, at times (46%), an H-bond with W161­(ACR) (Figure 2C and Table S6), as well as van der Waals interactions with V531 and L535 (both ACR) (Figure 2C and Table S6). The mutated residues form van der Waals interactions similar to those in the WT complex. However, these mutations mostly disrupt the intersubunits H-bonds (Figures 2D–F, Table S6) [However, R725 does form intrasubunit H-bonds with E665 (CYFIP2) (Figure 2F).]. These conclusions are qualitatively similar to those obtained by docking.^26^

**Figure 2:**
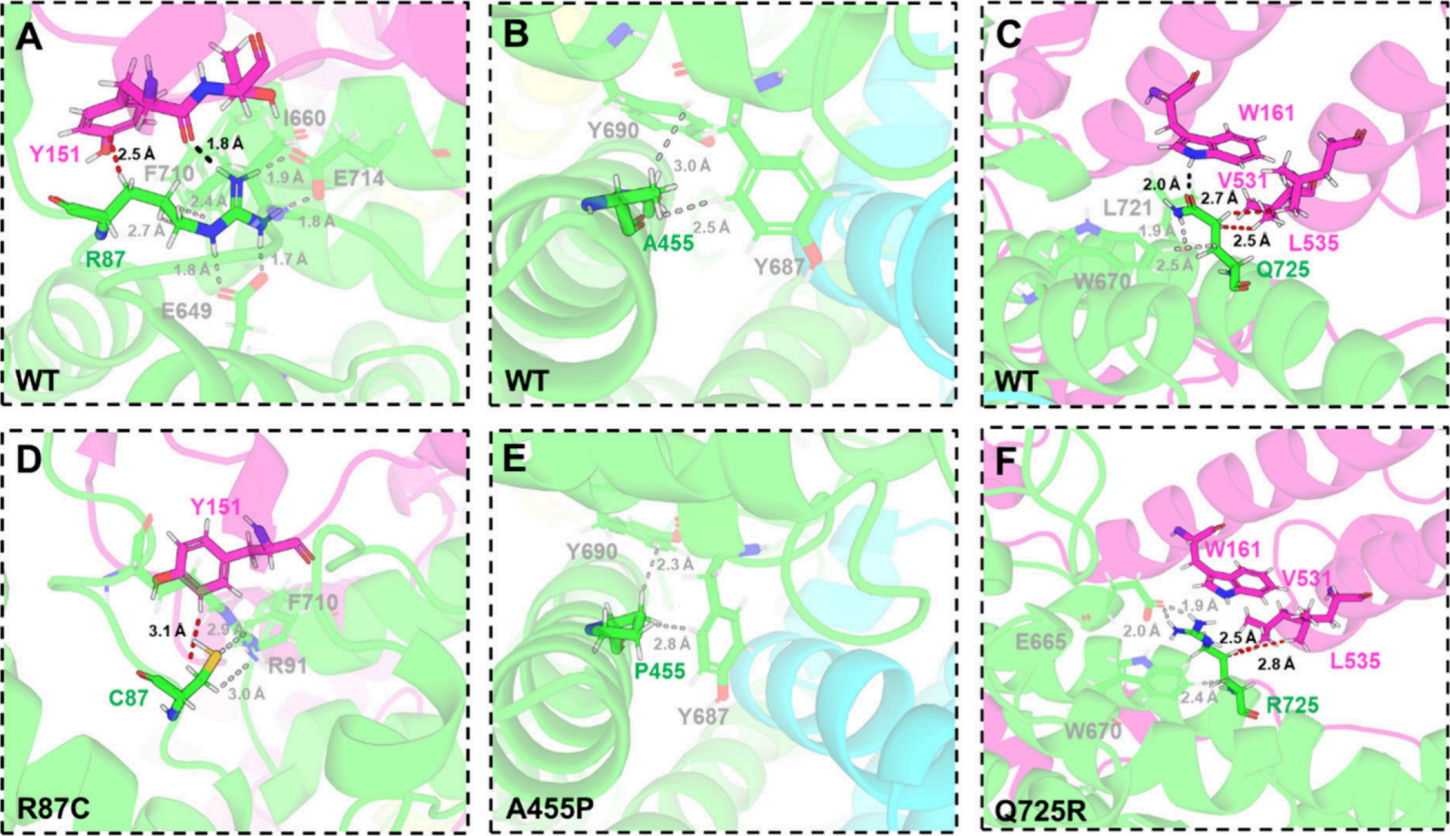
Alterations associated with the R87C (A,D), A455P (B,E), and Q725R (C,F) mutations in the chemical environment at the mutated sites. The models shown are representative MD structures (see Supporting Information (SI)). The color scheme is the same as in Figure 1. Residues involved in the mutation and their interacting groups are represented in sticks. H-bonds and van der Waals interactions are described as black and red dashed lines, respectively. Only the shortest van der Waals contacts are shown.

### ACR/WRC Interface Contacts

The mutants show a significant decrease (−17% to −12%) of the ACR/WRC contact areas when compared with the WT WRC (Figure 3A). The number of contacts (*Nc*) between non-hydrogen atoms in the ACR/WRC interface decreases consistently (Figure 3B), with Δ*Nc* ranging from −19% to −13%. In contrast, all of the other subunit/subunit interfaces show no specific trend (changes from −7% to +4% and Δ*Nc* spanning from −4% to +3%, Figure S6). Thus, despite being in different parts of the complex (Figure 1), the three mutations selectively weaken ACR interactions with the rest of the complex without significantly affecting the other contacts. This may facilitate ACR detachment (Figures 1 and S1) even in the absence of Rac1.^19^

**Figure 3:**
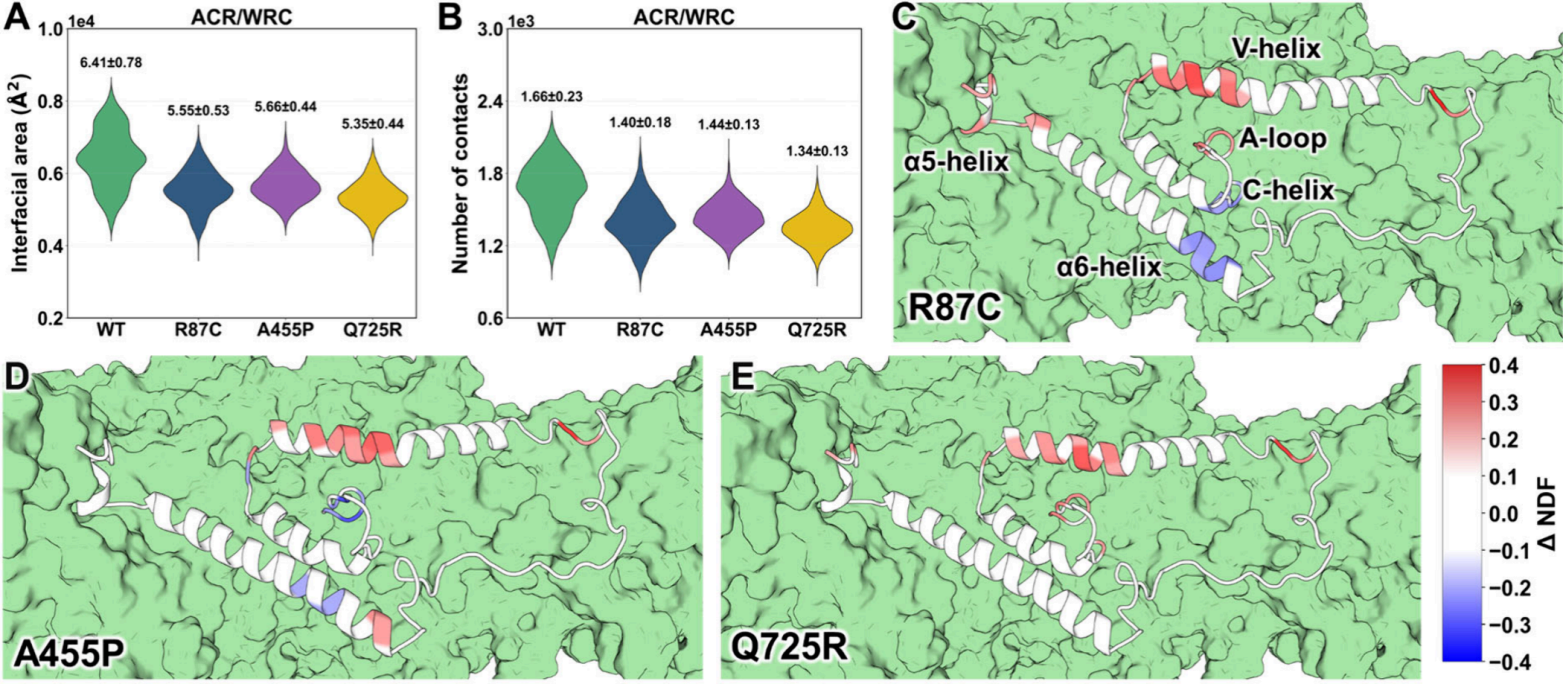
The effect of R87C, A455P, and Q725R mutations on the interactions between ACR and the rest of WRC. (A) ACR/WRC interfacial areas. (B) Number of heavy-atom contacts within 5 Å (*Nc*). (C–E) ΔNDF_i_ values for the ACR residues. They range from −0.4 (blue) to +0.4 (red). The ACR is represented as a cartoon, the rest as a green surface. Data for the entire complex are shown in Figure S7.

### ARC Destabilization

The normalized distance fluctuations score for residue *i* ^27,28^ (NDF_i_) provides information on the correlation of the residue’s motion with the rest of the protein. Positive scores of the difference between variants and WT (ΔNDF_i_; variant minus WT) are associated with increased disorder for the residue *i*.^29–34^ The opposite holds true for negative values. Most ACR residues in the ACR loops and the C-helix exhibit small values of |ΔNDF_i_| (0.1 or less, Figure 3C–E), while the ΔNDF_i_ of part of the V-helix (residues 514–525, Figure 1) ranges between 0.11 and 0.27 for the three mutants, suggesting that this segment may unfold more efficiently than the WT upon mutations.^29–33^ Some parts of the α6 helix in the R87C and A455P variants are prone to unfolding or stiffening, respectively. Similarly, the α5 helix tends to unfold in R87C. The loop regions show variant-dependent complex changes (Figure 3C–E). In summary, the mutations appear to predominantly destabilize the V-helix, while most parts of ACR, as well as the rest of the WRC, are not primarily affected (Figure S7).

### Large-Scale Motions

An analysis of the dynamic cross-correlation matrix suggests a domain-dependent motion in the WT complex (Figure 4A). ACR anticorrelates with the N-terminal halves and correlates with the C-terminal halves of other subunits, respectively (Figure 4A). The mutations alter the motion correlation in a highly intricate (albeit rather similar) manner (Figure 4B–D; Tables S7–S8): pairwise comparisons of the variant-induced different motion correlation matrices yield both cosine similarity and a Spearman correlation of approximately 0.5. The three largest eigenvectors of the WT and variants, obtained by principal component analysis, account for about 50% of the total motion (Figure S8). Interestingly, they involve ACR in the WT, and more extensively in the variants (20%, 33%, 22%, and 48%, for WT, R87C, A455P, and Q725R WRC, respectively) [Also, a small loop of NCKAP1 in Figure S8 shows large-scale motions. Its total contribution is 5% or less. The time scale investigated (6 μs) affects the convergence of this result. Thus, we discuss these findings only qualitatively]. Such larger-scale motions may facilitate ACR detachment.

**Figure 4:**
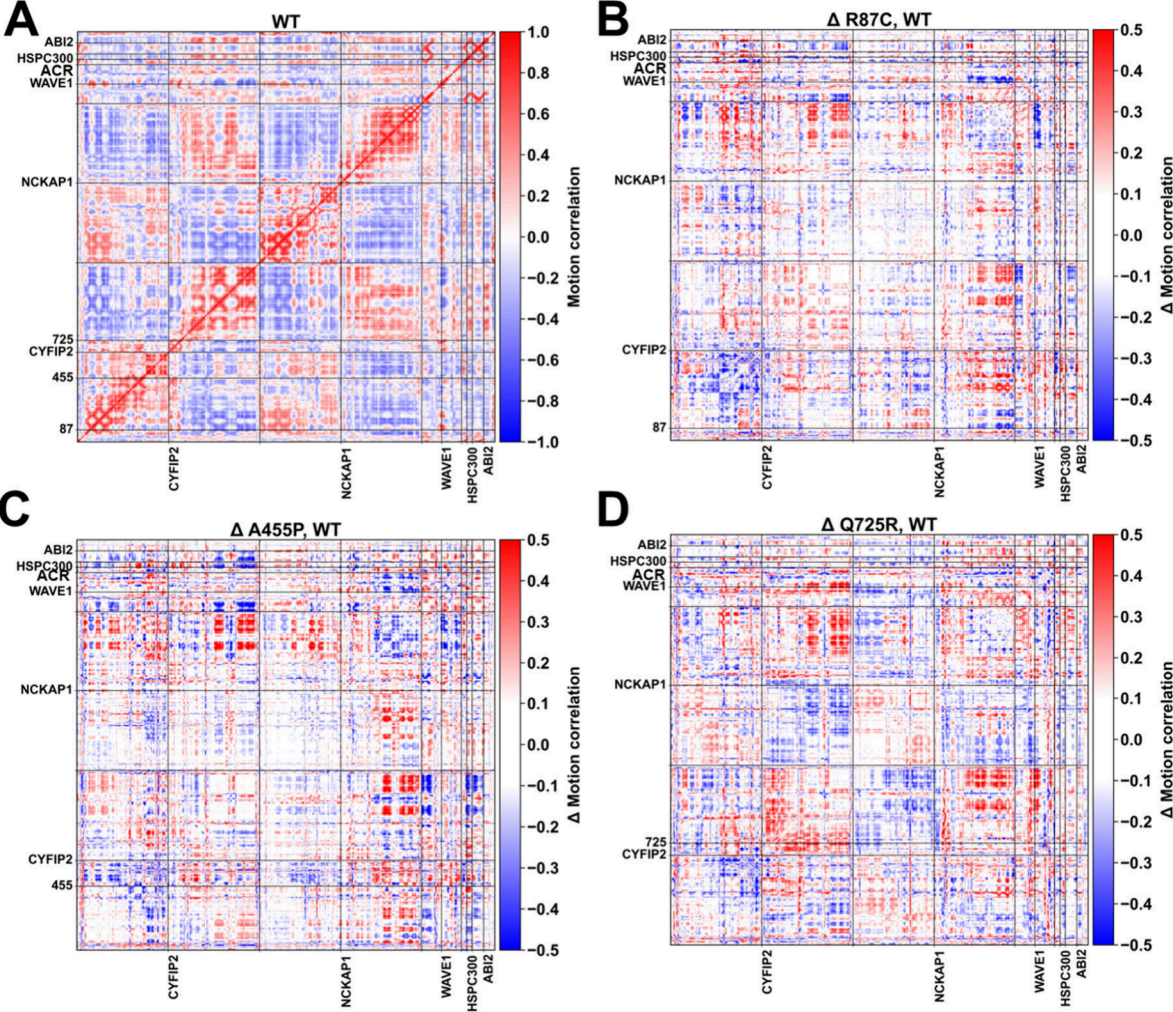
The effect of mutations on the motion correlation. (A) The motion correlation matrix for WT WRC and (B,D) the differences in motion correlation matrices between variants (R87C, A455P, and Q725R) and the WT.

ASD-linked missense mutations can alter protein–protein interactions (PPIs) up to 25%.^35,36^ Table S9 reports mutations known to affect structure or affinity among PPIs in vitro. All of them affect the binding of cellular partners via mechanisms that remain to be fully elucidated.^35–42^ Our study provides a new role of ASD-linked mutations, namely, to affect the binding of two subunits, CYFIP2 and WAVE1, within the same complex.

## 3 Conclusion

We investigated the impact of three NDD-linked variants (A455P, R87C, and Q725R) on the structural dynamics of the WRC inactive form by biomolecular simulations. We focused on the ACR, which undergoes conformational rearrangements during aberrant activation of the variants.

The first mutation is internal, while the others are at the ACR/CYFIP2 interface (Figure 1). All of them weaken the interactions between the ACR and the rest of the WRC (Figure 3A,B). This result could be expected by visual inspection for the R87C and Q725R WRC as the mutations directly affect the ACR/CYFIP2 interface (Figure 1), consistent with computations^26,43^ and GST pull-down experiments.^21^ Indeed, they disrupt interfacial H-bonds with ACR at the mutant sites while maintaining their van der Waals interactions (Figure 2 and Table S6). However, it is intriguing that subunit–subunit interactions are similarly affected by the A455P mutation: here, the changes in interactions lead to an allosteric change at the ACR/WRC surface, particularly the ACR/CYFIP2 interface (Table S10).

Strikingly, all mutations increase the propensity for unfolding in the V-helix of ACR (Figure 3C–E), besides affecting helices α5, α6, and C to different extents. The mutations also alter large-scale motions of the entire complex by involving ACR more than WT WRC (Figure S8). Such motions could play a role in ACR detachment from the rest of the complex. The similarity of effects across the mutations leads us to suggest that the way A455P can affect aberrant WRC activation is similar to that of the other two variants.^19^

Our findings suggest that the R87C, Q725R, and A455P mutations facilitate the detachment of ACR, even if interactors such as Rac1 do not bind to the WRC. We suggest here that the detachment occurs via an allosteric mechanism in the case of A455P (Figures 1 and 3), and via destabilization of ACR/CYFIP2 interactions in the case of R87C and Q725R (Figures 2 and 3). The resulting aberrant activation of the WRC may lead to deregulated downstream actin remodeling.^19^ Ultimately, this process leads to synaptic and spine morphological abnormalities,^15^ resulting in NDDs, including ASD.^20,44^

Based on these findings, we propose that ligands stabilizing the ACR/CYFIP2 interface or the V-helix might assist the restoration of normal WRC regulation for the mutants studied here.

## Supplementary Material



## Data Availability

Data, including input, parameter, and analysis script files for the MD simulations, can be found in the Zenodo repository: https://zenodo.org/records/15478773

## Notes

Summary of the setup details of MD simulations and post-MD analysis; schematic of WRC active form; convergence assessment of MD simulations; interfacial areas and number of heavy-atom contacts for other subunit/subunit interfaces; Δ*NDF*_*i*_ analysis for entire WRC; principal component analysis; known NDDs-linked variants of CYFIP2; predicted pathogenicity; system information; H-bonds and contact scores; cosine and Spearman correlation similarity; known mutations affect protein–protein interactions in ASD; largest changes in contact scores (PDF)

## Abbreviations

WRC: WAVE regulatory complex
NDDs: neurodevelopmental disorders
ASD: autism spectrum disorder
ACR: active C-terminal region
CYFIP2: cytoplasmic FMR1-Interacting protein 2
NCKAP1: non-catalytic region of tyrosine kinase associated protein 1
VCA: A-loop, V- and C-helices
MD: molecular dynamics
WT: wild-type
SI: Supporting Information
Nc: number of contacts
NDF_i_: normalized distance fluctuations score for residue i. PPIs, protein–protein interactions

## References

[1] Thapar A., Rutter M. (2021). Genetic Advances in Autism. Journal
of Autism and Developmental Disorders.

[2] Lord C., Brugha T. S., Charman T., Cusack J., Dumas G., Frazier T., Jones E. J. H., Jones R. M., Pickles A., State M. W., Taylor J. L., Veenstra-VanderWeele J. (2020). Autism spectrum
disorder. Nature Reviews Disease Primers.

[3] Zeidan J., Fombonne E., Scorah J., Ibrahim A., Durkin M. S., Saxena S., Yusuf A., Shih A., Elsabbagh M. (2022). Global prevalence
of autism: A systematic review update. Autism
Research.

[4] Guthrie W., Swineford L. B., Nottke C., Wetherby A. M. (2013). Early diagnosis
of autism spectrum disorder: stability and change in clinical diagnosis
and symptom presentation. Journal of Child Psychology
and Psychiatry.

[5] Willsey H. R., Willsey A. J., Wang B., State M. W. (2022). Genomics, convergent
neuroscience and progress in understanding autism spectrum disorder. Nat. Rev. Neurosci..

[6] Gaugler T., Klei L., Sanders S. J., Bodea C. A., Goldberg A. P., Lee A. B., Mahajan M., Manaa D., Pawitan Y., Reichert J., Ripke S., Sandin S., Sklar P., Svantesson O., Reichenberg A., Hultman C. M., Devlin B., Roeder K., Buxbaum J. D. (2014). Most genetic
risk for autism resides
with common variation. Nat. Genet..

[7] De
Rubeis S., He X., Goldberg A. P., Poultney C. S., Samocha K., Ercument Cicek A., Kou Y., Liu L., Fromer M., Walker S., Singh T., Klei L., Kosmicki J., Fu S.-C., Aleksic B., Biscaldi M., Bolton P. F., Brownfeld J. M., Cai J., Campbell N. G., Carracedo A., Chahrour M. H., Chiocchetti A. G., Coon H., Crawford E. L., Crooks L., Curran S. R., Dawson G., Duketis E., Fernandez B. A., Gallagher L., Geller E., Guter S. J., Sean
Hill R., Ionita-Laza I., Jimenez Gonzalez P., Kilpinen H., Klauck S. M., Kolevzon A., Lee I., Lei J., Lehtimäki T., Lin C.-F., Ma’ayan A., Marshall C. R., McInnes A. L., Neale B., Owen M. J., Ozaki N., Parellada M., Parr J. R., Purcell S., Puura K., Rajagopalan D., Rehnström K., Reichenberg A., Sabo A., Sachse M., Sanders S. J., Schafer C., Schulte-Rüther M., Skuse D., Stevens C., Szatmari P., Tammimies K., Valladares O., Voran A., Wang L.-S., Weiss L. A., Jeremy Willsey A., Yu T. W., Yuen R. K. C., Cook E. H., Freitag C. M., Gill M., Hultman C. M., Lehner T., Palotie A., Schellenberg G. D., Sklar P., State M. W., Sutcliffe J. S., Walsh C. A., Scherer S. W., Zwick M. E., Barrett J. C., Cutler D. J., Roeder K., Devlin B., Daly M. J., Buxbaum J. D. (2014). Synaptic, transcriptional and chromatin
genes disrupted in autism. Nature.

[8] Fu J. M., Satterstrom F. K., Peng M., Brand H., Collins R. L., Dong S., Wamsley B., Klei L., Wang L., Hao S. P., Stevens C. R., Cusick C., Babadi M., Banks E., Collins B., Dodge S., Gabriel S. B., Gauthier L., Lee S. K., Liang L., Ljungdahl A., Mahjani B., Sloofman L., Smirnov A. N., Barbosa M., Betancur C., Brusco A., Chung B. H. Y., Cook E. H., Cuccaro M. L., Domenici E., Ferrero G. B., Gargus J. J., Herman G. E., Hertz-Picciotto I., Maciel P., Manoach D. S., Passos-Bueno M. R., Persico A. M., Renieri A., Sutcliffe J. S., Tassone F., Trabetti E., Campos G., Cardaropoli S., Carli D., Chan M. C. Y., Fallerini C., Giorgio E., Girardi A. C., Hansen-Kiss E., Lee S. L., Lintas C., Ludena Y., Nguyen R., Pavinato L., Pericak-Vance M., Pessah I. N., Schmidt R. J., Smith M., Costa C. I. S., Trajkova S., Wang J. Y. T., Yu M. H. C., Aleksic B., Artomov M., Benetti E., Biscaldi-Schafer M., Børglum A. D., Carracedo A., Chiocchetti A. G., Coon H., Doan R. N., Fernández-Prieto M., Freitag C. M., Gerges S., Guter S., Hougaard D. M., Hultman C. M., Jacob S., Kaartinen M., Kolevzon A., Kushima I., Lehtimäki T., Rizzo C. L., Maltman N., Manara M., Meiri G., Menashe I., Miller J., Minshew N., Mosconi M., Ozaki N., Palotie A., Parellada M., Puura K., Reichenberg A., Sandin S., Scherer S. W., Schlitt S., Schmitt L., Schneider-Momm K., Siper P. M., Suren P., Sweeney J. A., Teufel K., del Pilar Trelles M., Weiss L. A., Yuen R., Cutler D. J., De Rubeis S., Buxbaum J. D., Daly M. J., Devlin B., Roeder K., Sanders S. J., Talkowski M. E. (2022). Rare coding
variation provides insight into the genetic architecture and phenotypic
context of autism. Nat. Genet..

[9] Zweier M., Begemann A., McWalter K., Cho M. T., Abela L., Banka S., Behring B., Berger A., Brown C. W., Carneiro M., Chen J., Cooper G. M., Finnila C. R., Guillen Sacoto M. J., Henderson A., Hüffmeier U., Joset P., Kerr B., Lesca G., Leszinski G. S., McDermott J. H., Meltzer M. R., Monaghan K. G., Mostafavi R., Õunap K., Plecko B., Powis Z., Purcarin G., Reimand T., Riedhammer K. M., Schreiber J. M., Sirsi D., Wierenga K. J., Wojcik M. H., Papuc S. M., Steindl K., Sticht H., Rauch A. (2019). Spatially clustering
de novo variants in CYFIP2, encoding the cytoplasmic FMRP interacting
protein 2, cause intellectual disability and seizures. European Journal of Human Genetics.

[10] Mariano V., Kanellopoulos A. K., Ricci C., Di Marino D., Borrie S. C., Dupraz S., Bradke F., Achsel T., Legius E., Odent S., Billuart P., Bienvenu T., Bagni C. (2024). Intellectual Disability
and Behavioral Deficits Linked to *CYFIP1* Missense
Variants Disrupting Actin Polymerization. Biol.
Psychiatry.

[11] Guo H., Zhang Q., Dai R., Yu B., Hoekzema K., Tan J., Tan S., Jia X., Chung W. K., Hernan R., Alkuraya F. S., Alsulaiman A., Al-Muhaizea M. A., Lesca G., Pons L., Labalme A., Laux L., Bryant E., Brown N. J., Savva E., Ayres S., Eratne D., Peeters H., Bilan F., Letienne-Cejudo L., Gilbert-Dussardier B., Ruiz-Arana I.-L., Merlini J. M., Boizot A., Bartoloni L., Santoni F., Karlowicz D., McDonald M., Wu H., Hu Z., Chen G., Ou J., Brasch-Andersen C., Fagerberg C. R., Dreyer I., chun-hui Tsai A., Slegesky V., McGee R. B., Daniels B., Sellars E. A., Carpenter L. A., Schaefer B., Sacoto M. J. G., Begtrup A., Schnur R. E., Punj S., Wentzensen I. M., Rhodes L., Pan Q., Bernier R. A., Chen C., Eichler E. E., Xia K. (2020). *NCKAP1* Disruptive
Variants Lead to a Neurodevelopmental Disorder with Core
Features of Autism. American Journal of Human
Genetics.

[12] Chen Z., Borek D., Padrick S. B., Gomez T. S., Metlagel Z., Ismail A. M., Umetani J., Billadeau D. D., Otwinowski Z., Rosen M. K. (2010). Structure and control of the actin
regulatory WAVE complex. Nature.

[13] Rottner K., Stradal T. E. B., Chen B. (2021). WAVE regulatory
complex. Curr. Biol..

[14] Chen B., Brinkmann K., Chen Z., Pak C. W., Liao Y., Shi S., Henry L., Grishin N. V., Bogdan S., Rosen M. K. (2014). The WAVE
Regulatory Complex Links Diverse Receptors to the Actin Cytoskeleton. Cell.

[15] De
Rubeis S., Pasciuto E., Li Ka W., Fernández E., Di Marino D., Buzzi A., Ostroff L. E., Klann E., Zwartkruis F. J. T., Komiyama N. H., Grant S. G. N., Poujol C., Choquet D., Achsel T., Posthuma D., Smit A. B., Bagni C. (2013). CYFIP1 Coordinates mRNA Translation and Cytoskeleton Remodeling to
Ensure Proper Dendritic Spine Formation. Neuron.

[16] Hsiao K., Harony-Nicolas H., Buxbaum J. D., Bozdagi-Gunal O., Benson D. L. (2016). Cyfip1 Regulates
Presynaptic Activity during Development. J.
Neurosci..

[17] Chen B., Chou H.-T., Brautigam C. A., Xing W., Yang S., Henry L., Doolittle L. K., Walz T., Rosen M. K. (2017). Rac1 GTPase
activates the WAVE regulatory complex through two distinct binding
sites. eLife.

[18] Ding B., Yang S., Schaks M., Liu Y., Brown A. J., Rottner K., Chowdhury S., Chen B. (2022). Structures reveal a
key mechanism of WAVE regulatory complex activation by Rac1 GTPase. Nat. Commun..

[19] Schaks M., Reinke M., Witke W., Rottner K. (2020). Molecular Dissection
of Neurodevelopmental Disorder-Causing Mutations in CYFIP2. Cells.

[20] Davenport E. C., Szulc B. R., Drew J., Taylor J., Morgan T., Higgs N. F., López-Doménech G., Kittler J. T. (2019). Autism and Schizophrenia-Associated CYFIP1 Regulates
the Balance of Synaptic Excitation and Inhibition. Cell Reports.

[21] Nakashima M., Kato M., Aoto K., Shiina M., Belal H., Mukaida S., Kumada S., Sato A., Zerem A., Lerman-Sagie T., Lev D., Leong H. Y., Tsurusaki Y., Mizuguchi T., Miyatake S., Miyake N., Ogata K., Saitsu H., Matsumoto N. (2018). De novo hotspot
variants in CYFIP2
cause early-onset epileptic encephalopathy. Ann. Neurol..

[22] López-Ferrando V., Gazzo A., de la Cruz X., Orozco M., Gelpí J. L. (2017). PMut: a
web-based tool for the annotation of pathological variants on proteins,
2017 update. Nucleic Acids Res..

[23] Pejaver V., Urresti J., Lugo-Martinez J., Pagel K. A., Lin G. N., Nam H.-J., Mort M., Cooper D. N., Sebat J., Iakoucheva L. M., Mooney S. D., Radivojac P. (2020). Inferring
the molecular and phenotypic impact of amino acid variants with MutPred2. Nat. Commun..

[24] Han K. A., Ko J. (2023). Orchestration of synaptic
functions by WAVE regulatory complex-mediated
actin reorganization. Experimental & Molecular
Medicine.

[25] Abramson J., Adler J., Dunger J., Evans R., Green T., Pritzel A., Ronneberger O., Willmore L., Ballard A. J., Bambrick J., Bodenstein S. W., Evans D. A., Hung C.-C., O’Neill M., Reiman D., Tunyasuvunakool K., Wu Z., Žemgulytė A., Arvaniti E., Beattie C., Bertolli O., Bridgland A., Cherepanov A., Congreve M., Cowen-Rivers A. I., Cowie A., Figurnov M., Fuchs F. B., Gladman H., Jain R., Khan Y. A., Low C. M. R., Perlin K., Potapenko A., Savy P., Singh S., Stecula A., Thillaisundaram A., Tong C., Yakneen S., Zhong E. D., Zielinski M., Žídek A., Bapst V., Kohli P., Jaderberg M., Hassabis D., Jumper J. M. (2024). Accurate structure prediction of
biomolecular interactions with AlphaFold 3. Nature.

[26] Xie S., Zuo K., De Rubeis S., Ruggerone P., Carloni P. (2025). Molecular basis of
the CYFIP2 and NCKAP1 autism-linked variants in the WAVE regulatory
complex. Protein Sci..

[27] Morra G., Potestio R., Micheletti C., Colombo G. (2012). Corresponding Functional
Dynamics across the Hsp90 Chaperone Family: Insights from a Multiscale
Analysis of MD Simulations. PLOS Computational
Biology.

[28] Frasnetti E., Cucchi I., Pavoni S., Frigerio F., Cinquini F., Serapian S. A., Pavarino L. F., Colombo G. (2024). Integrating Molecular
Dynamics and Machine Learning Algorithms to Predict the Functional
Profile of Kinase Ligands. J. Chem. Theory Comput..

[29] Torielli L., Guarra F., Shao H., Gestwicki J. E., Serapian S. A., Colombo G. (2025). Pathogenic mutation
impairs functional
dynamics of Hsp60 in mono- and oligomeric states. Nat. Commun..

[30] Triveri A., Casali E., Frasnetti E., Doria F., Frigerio F., Cinquini F., Pavoni S., Moroni E., Marchetti F., Serapian S. A., Colombo G. (2023). Conformational
Behavior of SARS-Cov-2
Spike Protein Variants: Evolutionary Jumps in Sequence Reverberate
in Structural Dynamic Differences. J. Chem.
Theory Comput..

[31] Moroni E., Agard D. A., Colombo G. (2018). The Structural Asymmetry
of Mitochondrial
Hsp90 (Trap1) Determines Fine Tuning of Functional Dynamics. J. Chem. Theory Comput..

[32] Rehn A., Moroni E., Zierer B. K., Tippel F., Morra G., John C., Richter K., Colombo G., Buchner J. (2016). Allosteric
Regulation Points Control the Conformational Dynamics of the Molecular
Chaperone Hsp90. J. Mol. Biol..

[33] Castelli M., Magni A., Bonollo G., Pavoni S., Frigerio F., Oliveira A. S. F., Cinquini F., Serapian S. A., Colombo G. (2024). Molecular
mechanisms of chaperone-directed protein folding: Insights from atomistic
simulations. Protein Sci..

[34] De
Marco M., Rai S. R., Scietti L., Mattoteia D., Liberi S., Moroni E., Pinnola A., Vetrano A., Iacobucci C., Santambrogio C., Colombo G., Forneris F. (2025). Molecular
structure and enzymatic mechanism of the human collagen hydroxylysine
galactosyltransferase GLT25D1/COLGALT1. Nat.
Commun..

[35] Chen S., Wang J., Cicek E., Roeder K., Yu H., Devlin B. (2020). De novo missense variants
disrupting protein-protein
interactions affect risk for autism through gene co-expression and
protein networks in neuronal cell types. Molecular
Autism.

[36] Chen S., Fragoza R., Klei L., Liu Y., Wang J., Roeder K., Devlin B., Yu H. (2018). An interactome
perturbation
framework prioritizes damaging missense mutations for developmental
disorders. Nat. Genet..

[37] Mameza M. G., Dvoretskova E., Bamann M., Hönck H.-H., Güler T., Boeckers T. M., Schoen M., Verpelli C., Sala C., Barsukov I., Dityatev A., Kreienkamp H.-J. (2013). SHANK3
Gene Mutations Associated with Autism Facilitate Ligand Binding to
the Shank3 Ankyrin Repeat Region*. J. Biol.
Chem..

[38] Stephenson J. R., Wang X., Perfitt T. L., Parrish W. P., Shonesy B. C., Marks C. R., Mortlock D. P., Nakagawa T., Sutcliffe J. S., Colbran R. J. (2017). A Novel Human *CAMK2A* Mutation Disrupts
Dendritic Morphology and Synaptic Transmission, and Causes ASD-Related
Behaviors. J. Neurosci..

[39] Taketomi T., Yasuda T., Morita R., Kim J., Shigeta Y., Eroglu C., Harada R., Tsuruta F. (2022). Autism-associated
mutation
in Hevin/Sparcl1 induces endoplasmic reticulum stress through structural
instability. Sci. Rep..

[40] Mohamed M. S., Klann E. (2023). Autism- and epilepsy-associated EEF1A2
mutations lead to translational
dysfunction and altered actin bundling. Proc.
Natl. Acad. Sci. U. S. A..

[41] Ascano M., Mukherjee N., Bandaru P., Miller J. B., Nusbaum J. D., Corcoran D. L., Langlois C., Munschauer M., Dewell S., Hafner M., Williams Z., Ohler U., Tuschl T. (2012). FMRP targets distinct mRNA sequence elements to regulate
protein expression. Nature.

[42] Li J., Ma Z., Shi M., Malty R. H., Aoki H., Minic Z., Phanse S., Jin K., Wall D. P., Zhang Z., Urban A. E., Hallmayer J., Babu M., Snyder M. (2015). Identification
of Human Neuronal Protein Complexes Reveals Biochemical Activities
and Convergent Mechanisms of Action in Autism Spectrum Disorders. Cell Systems.

[43] Biembengut Í. V., Shigunov P., Frota N. F., Lourenzoni M. R., de Souza T. A. C. B. (2022). Molecular Dynamics of CYFIP2 Protein and Its R87C Variant
Related to Early Infantile Epileptic Encephalopathy. International Journal of Molecular Sciences.

[44] Pathania M., Davenport E. C., Muir J., Sheehan D. F., López-Doménech G., Kittler J. T. (2014). The autism and schizophrenia associated gene CYFIP1
is critical for the maintenance of dendritic complexity and the stabilization
of mature spines. Translational Psychiatry.

