# Supplementary material for: Impact of Genetic Variants Associated with Neurodevelopmental Disorders on the WAVE Regulatory Complex
Source: J Chem Inf Model. 2025 Jul 9;65(14):7399–405. doi: 10.1021/acs.jcim.5c01162 (PMC12308791; doi:10.1021/acs.jcim.5c01162)
Supplement: Supplementary file 1 [file ci5c01162_si_001.pdf]

Impact of genetic variants associated with neurodevelopmental disorders on the WAVE regulatory complex

Song Xie [a, b], *Ke Zuo* [a, c, and d*], *Silvia De Rubeis* [e, f, g, h, i, and j], *Giorgio Bonollo* [k], *Giorgio Colombo* [k], *Paolo Ruggerone* [d*], *and Paolo Carloni* [a, b, and l*]

[a] Computational Biomedicine, Institute of Neuroscience and Medicine INM-9, Forschungszentrum Jülich GmbH, Jülich, Germany.

[b] Department of Physics, RWTH Aachen University, Aachen, Germany.

[c] National & Local Joint Engineering Research Center of Targeted and Innovative Therapeutics, Chongqing Key Laboratory of Kinase Modulators as Innovative Medicine, College of Pharmacy (International Academy of Targeted Therapeutics and Innovation), Chongqing University of Arts and Sciences, Chongqing, China.

[d] Department of Physics, University of Cagliari, Monserrato, Cagliari, Italy

[e] Seaver Autism Center for Research and Treatment, Icahn School of Medicine at Mount Sinai, New York, USA

[f] Department of Psychiatry, Icahn School of Medicine at Mount Sinai, New York, USA.

[g] The Mindich Child Health and Development Institute, Icahn School of Medicine at Mount Sinai, New York, USA.

[h] Friedman Brain Institute, Icahn School of Medicine at Mount Sinai, New York, USA.

[i] Department of Pharmacological Sciences, Icahn School of Medicine at Mount Sinai, New York, USA.

[j] Alper Center for Neural Development and Regeneration Friedman Brain Institute, Icahn School of Medicine at Mount Sinai, New York, USA.

[k] Dipartimento di Chimica, Università di Pavia, Via Taramelli 12, Pavia, Italy

[l] JARA Institute: Molecular Neuroscience and Imaging, Institute of Neuroscience and Medicine INM-11, Forschungszentrum Jülich GmbH, Jülich, Germany

Keywords: WAVE regulatory complex, neurodevelopmental disorders, autism spectrum disorder, missense variants, molecular dynamics

* The corresponding author. P.C: p.carloni@fz-juelich.de; K. Z.: k.zuo@stimulate-ejd.eu; P. R.: paolo.ruggerone@unica.it

Supporting Method

Model Construction. We constructed the WT WRC isoform that exhibits the highest number of neurodevelopmental disorders (NDDs)-linked mutations [1] using AlphaFold 3 (AF3) [2]. WRC contains three and two NDD-linked isoforms for CYFIP2 and NCKAP1, respectively [1], while for the other subunits, no clear association links their specific isoforms to NDDs. Thus, we considered the isoforms of CYFIP2 and NCKAP1 with the highest number of NDDS-linked mutations as in ref. [1], and the isoforms of the others found in the crystal structure [3](**Table S4**).

The initial model of the WT WRC inactive form was then built using AlphaFold 3 [2]. Its predicted template modeling (pTM) and interface predicted Template Modeling (ipTM) scores [2] are both as high as 0.91. The Cα RMSD values between our model and the available X-ray[3, 4] and Cyro-EM structures[5] of WRC inactive form are 0.9 Å or less (**Table S5**).

R87C WRC, A455P WRC, and Q725R WRC were generated using the Swiss-Model web server [6]starting from WT WRC. The complexes consisted of 2,924 residues (**Table S4**).

The H++ web server [7] was used to determine the protonation states of Asp, Glu, Arg, Lys, and His residues at the physiological pH of 7.4.
Next, the complex was solvated in a truncated octahedral water box with a minimum solute-to-edge distance of 13 Å. Na^+ and Cl^- ions are added to neutralize the system. The resulting concentration of NaCl was 0.15 mM (**Table S3**). Periodic boundary conditions were applied.

MD simulations were carried out using the AMBER 22 software [8]. The AMBER ff19SB [9], OPC [10], and Åqvist [11] force fields were utilized for the proteins, water, and the Na^+ and Cl^- ions, respectively. Long-range electrostatic interactions were calculated by the Particle Mesh Ewald method [12]. A cutoff distance of 10 Å was applied to short-range non-bonded interactions, which include Lennard-Jones forces and the short-range component of the PME calculation.

The systems underwent three successive rounds of minimization: (i) 10,000 steps of steepest descent followed by 10,000 steps of conjugate gradient minimization with a 100 kcal/(mol·Å^2) restraint applied to the entire solute; (ii) the same protocol with the same restraints but applied only to the heavy atoms; and (iii) the same protocol without any restraints. The systems were then heated from 100 K to 310 K over 0.5 ns using Langevin dynamics [13]. A 100 kcal/(mol·Å^2) constraint on the heavy atoms was used. The systems then underwent another 0.5 ns at 310 K without restraints. An integration time step of 1 fs was used. Successively, each system underwent three independent 2 µs-long isobaric-isothermal simulations, each

initiated with different velocities. Temperature (310 K) and pressure (1 bar) were maintained using Langevin dynamics [13] and a Monte Carlo barostat [14], respectively. A time step of 2 fs was used.

Properties were calculated over 4.5 μs of equilibrated trajectories, compiled by pooling the final 1.5 μs from each of three independent replicas: (i) *Clustering of the MD structures.* This was performed on backbone RMSD values, using the DBSCAN algorithm [15]. The min-points = 5 and ε = 2.0 Å were set. (ii) *The radius of gyratio*n; (iii) The *interfacial contact area.* For the two subunits A and B, this reads:

$$SASA(A) + SASA(B) - SASA(AB) \qquad (1)$$

Where SASA is the solvent accessible surface area [16]; (iv) *The number of contacts*. This is defined by considering the non-hydrogen atoms with a cut-off of 5 Å. The residue-pair contact scores were calculated by dividing the total non-hydrogen atom contacts formed by the residue pair, summed across all frames, by the number of frames; (v) *The dynamic cross-correlation matrix* [17]; (vi) *The principal component vectors* [18]; (vii) *The H-bond occupancy.* This is defined as follows: An H-bond between a donor (D) and an acceptor (A) is formed if the distance between them is less than 3.2 Å and the corresponding D-H-A angle exceeds 125°. (viii) The *Distance Fluctuations* DF_{ij} [19] quantifies the correlation between the motion of residue pairs *i* and *j:*

$$DF_{ij} = \; < (d_{ij} -< d_{ij} >)^2 > \qquad (2)$$

Where d_{ij} represents the distance between Cα atoms of the residues in a particular MD frame, and < > denote averages over a whole trajectory.

The normalized DF_{ij} *value* reduces the impact of potential outlier values for pairs in loop regions:

$$NDF_{ij} = \frac{tDF_{ij}}{UB} \qquad (3)$$

UB (the upper bound) reads:

$$UB = Q3 + 1.5 \times \; (Q3 - Q1) \qquad (4)$$

Where Q1 and Q3 are the first and third quartiles [20] of DF_{ij}, respectively.

tDF_{ij} is the truncated DF_{ij} up to UB:

$$tDF_{ij} = \begin{cases} UB & if \; DF_{ij} \geq UB \\ DF_{ij} & if \; DF_{ij} \leq UB \end{cases} \qquad (5)$$

The normalizes DF_i *score* for residue *i* is obtained by averaging over the NDF_{ij}

$$NDF_i \; = \frac{1}{2924} \cdot (\textstyle\sum_1^{2924} NDF_{ij} \qquad (6)$$

The difference in NDF_i score (ΔNDF, hereafter) between WT and WRC variants

$$\Delta NDF_i = NDF_{i,mutant} - NDF_{i,WT} \qquad (7)$$

correlates with the efficiency of the folding/unfolding transitions [21, 22]. The higher the values, the more efficient the transitions in the residues involved [21, 22].

The CPPTRAJ code was used for (i)-(vii) [23], while the distance_fluctuation.py code [19] was used for (viii).

Supporting Figures

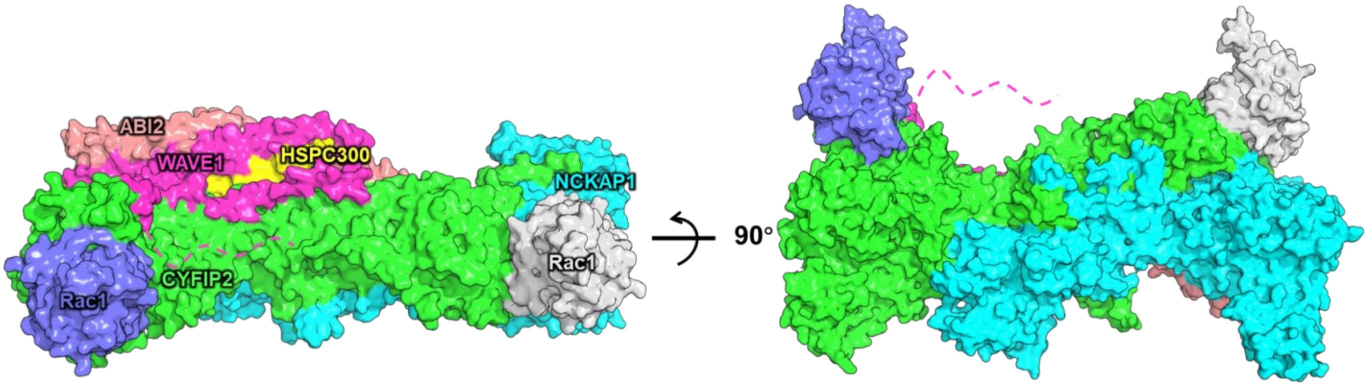

Figure S1. Schematic of WRC active form. The color scheme is the same as in **Figure 1**, with two additional Rac1 proteins binding to WRC. These are shown as blue and grey surfaces [5]. The model is based on the cryo-EM structure by Ding et al [5] (PDB ID: 7USE). The ACR is schematically represented by dashed lines to show its detachment, which is unfolded in the cryo-EM structure. No information on the structural determinants of the latter is provided.

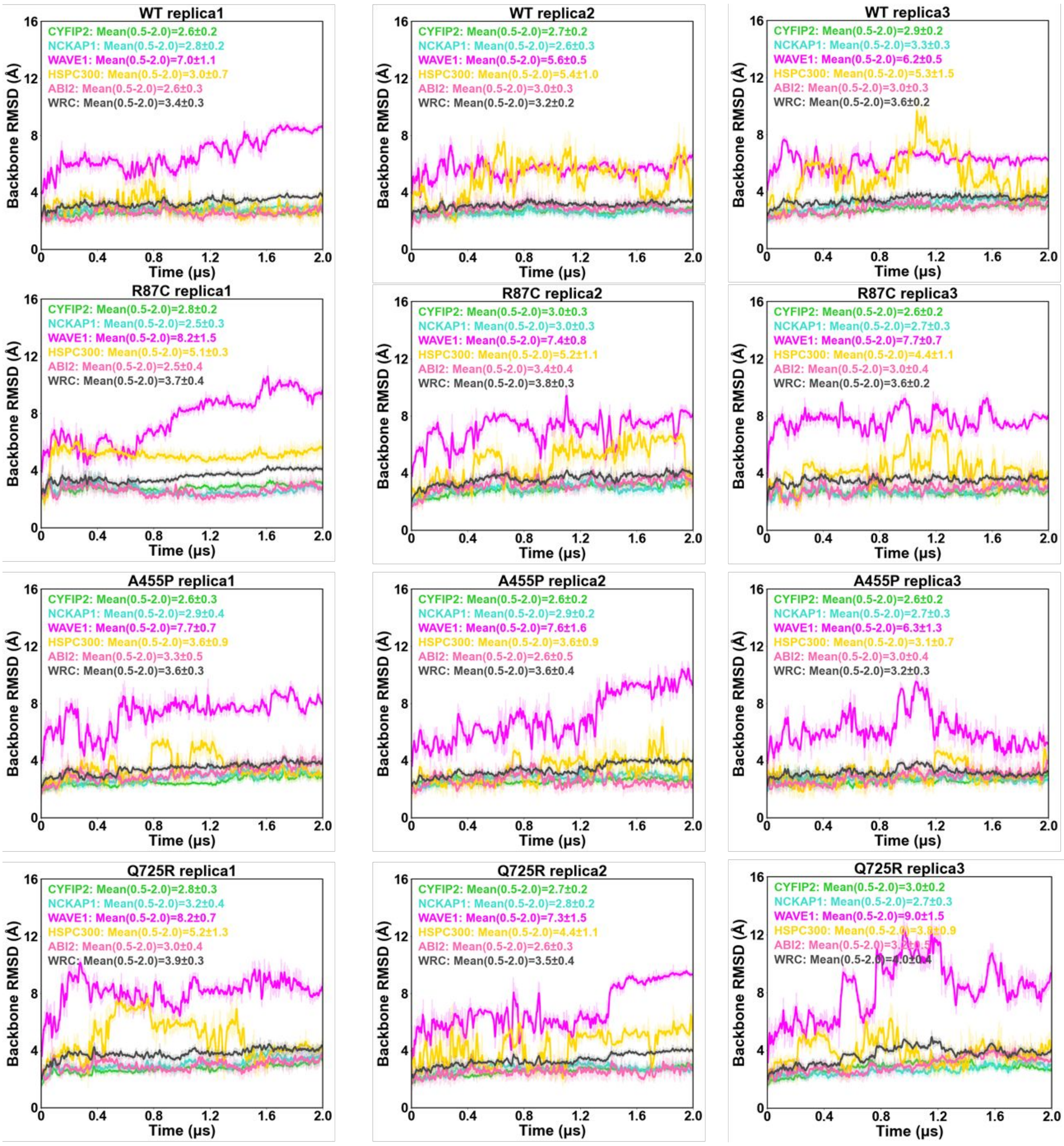

Figure S2. Backbone root-mean-square deviation (RMSD) of wild-type (WT) and NDDs-linked variants (R87C, A455P, Q725R) WRC plotted as a function of simulated time. Most RMSDs show fluctuations < 0.5 Å during the final 1.5 μs. As expected, the RMSD values for WAVE1 and HSPC300 flexible loos feature relatively large fluctuations in regions (see **Figures S3 and S4**). In contrast, the folded regions exhibit RMSD fluctuations below 0.5 Å (see **Figures S3 and S4**).

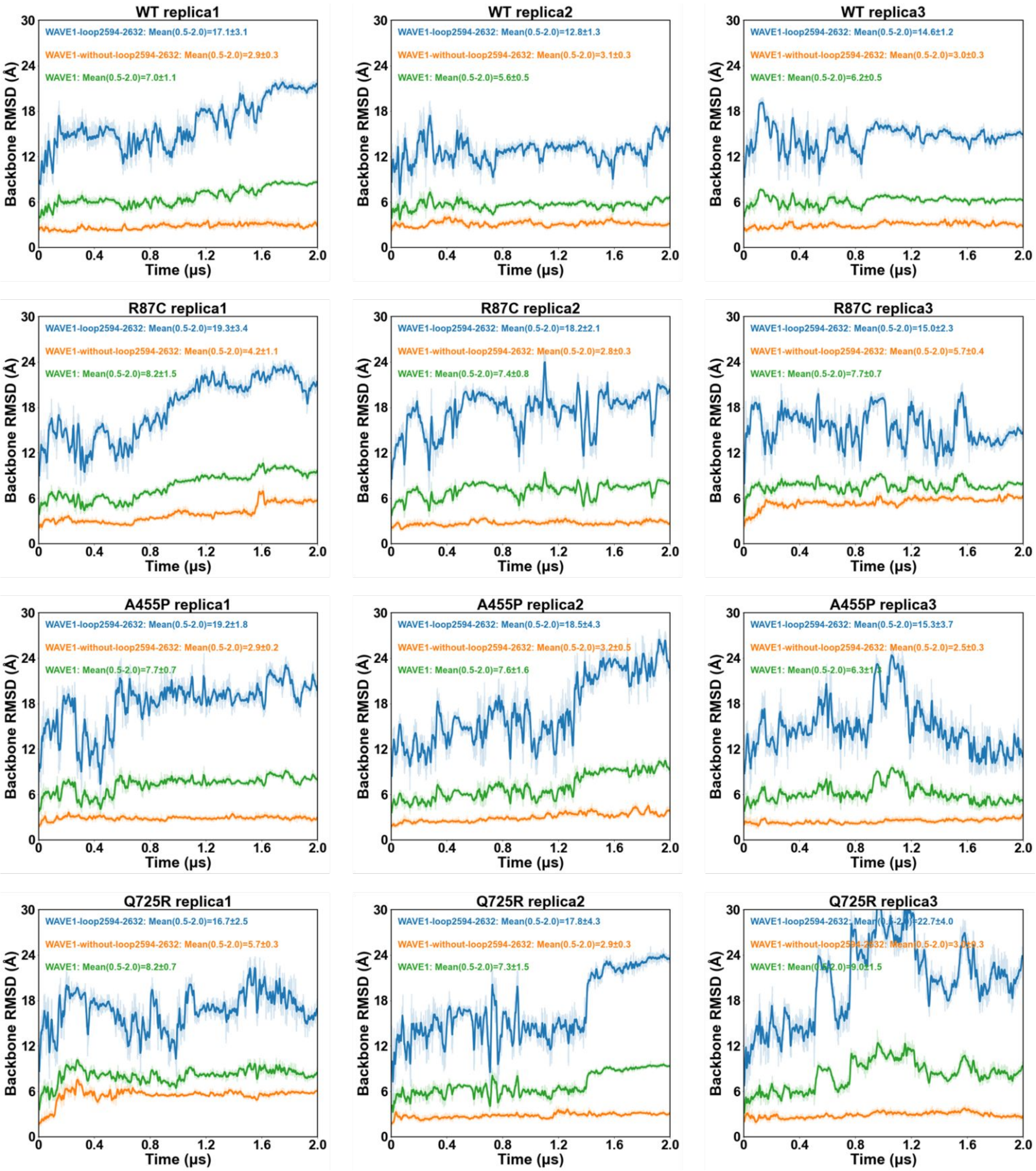

Figure S3. Same as **Figure S2** but for the WAVE1 protein.

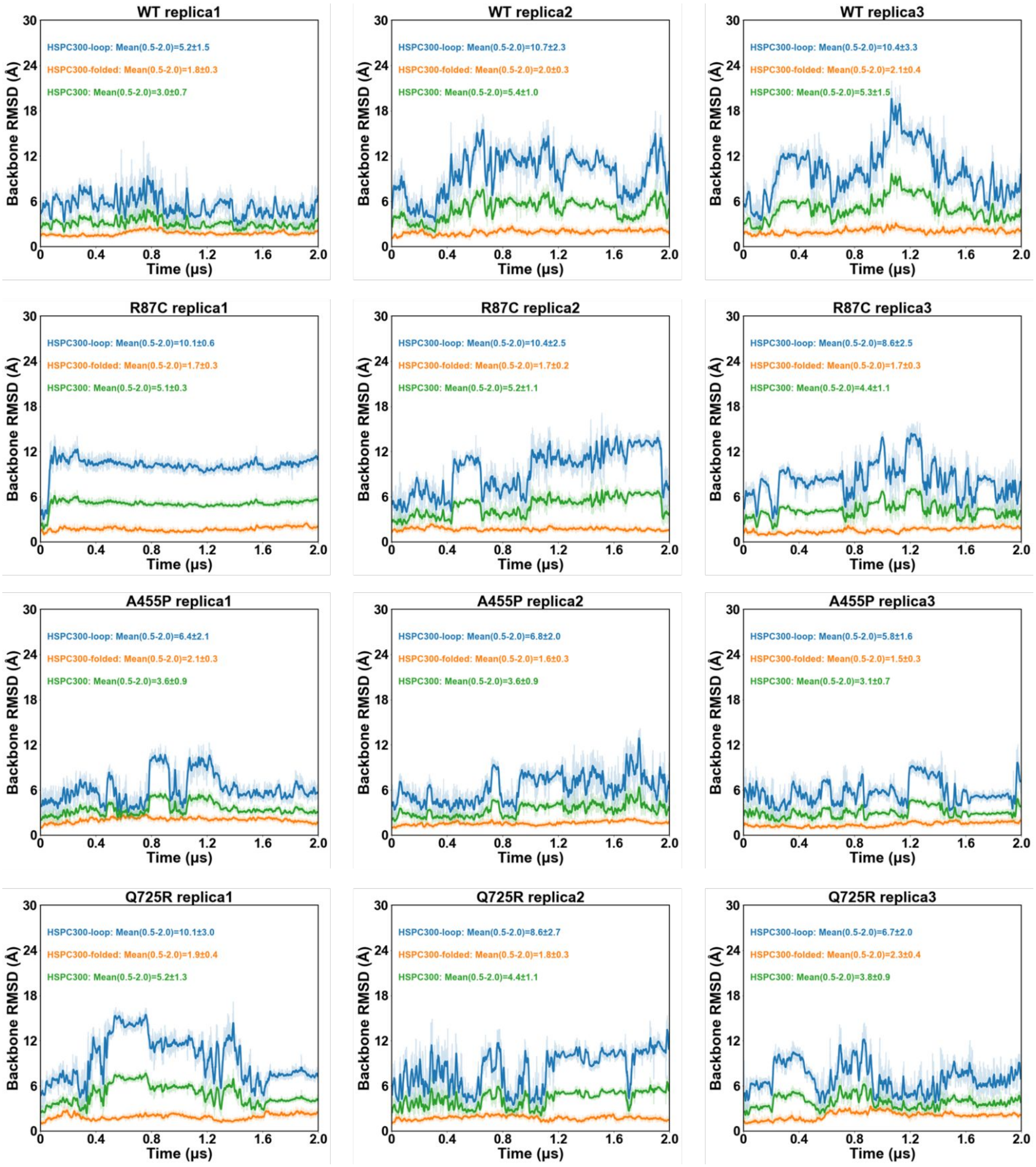

Figure S4. Same as **Figure S2** but for the HSPC300 protein.

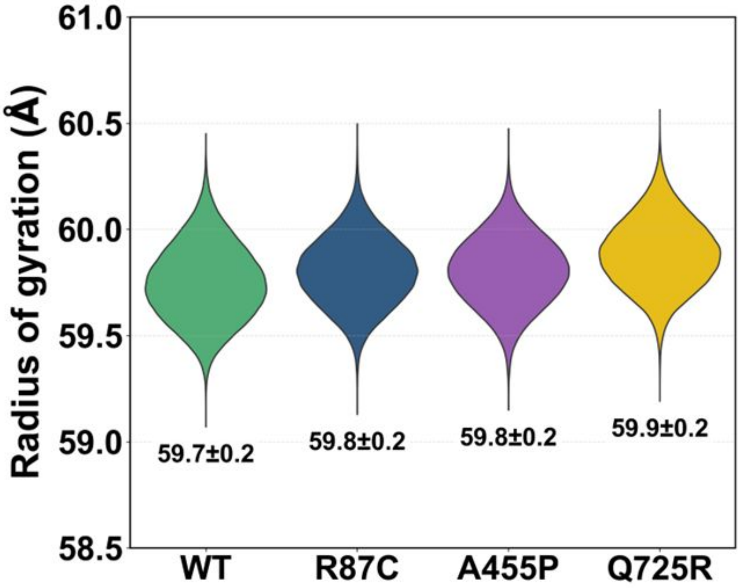

Figure S5. The radius of gyration of the four systems investigated here.

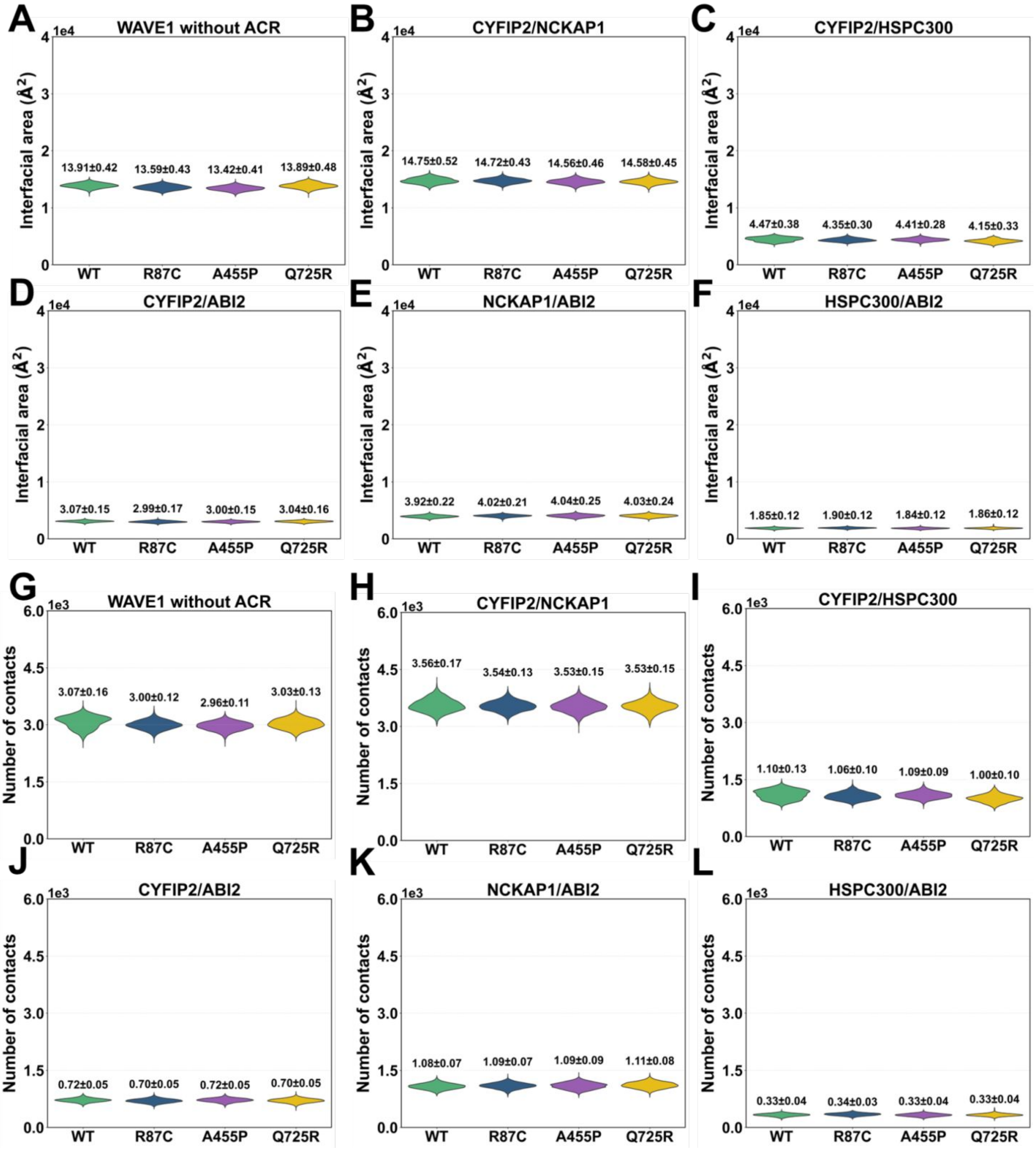

Figure S6. Interfacial areas (A-F) and number of heavy-atom contacts (G-L) of all protein/protein interfaces except for the ACR/WRC interface, which is shown in **Figures 2A and B**.

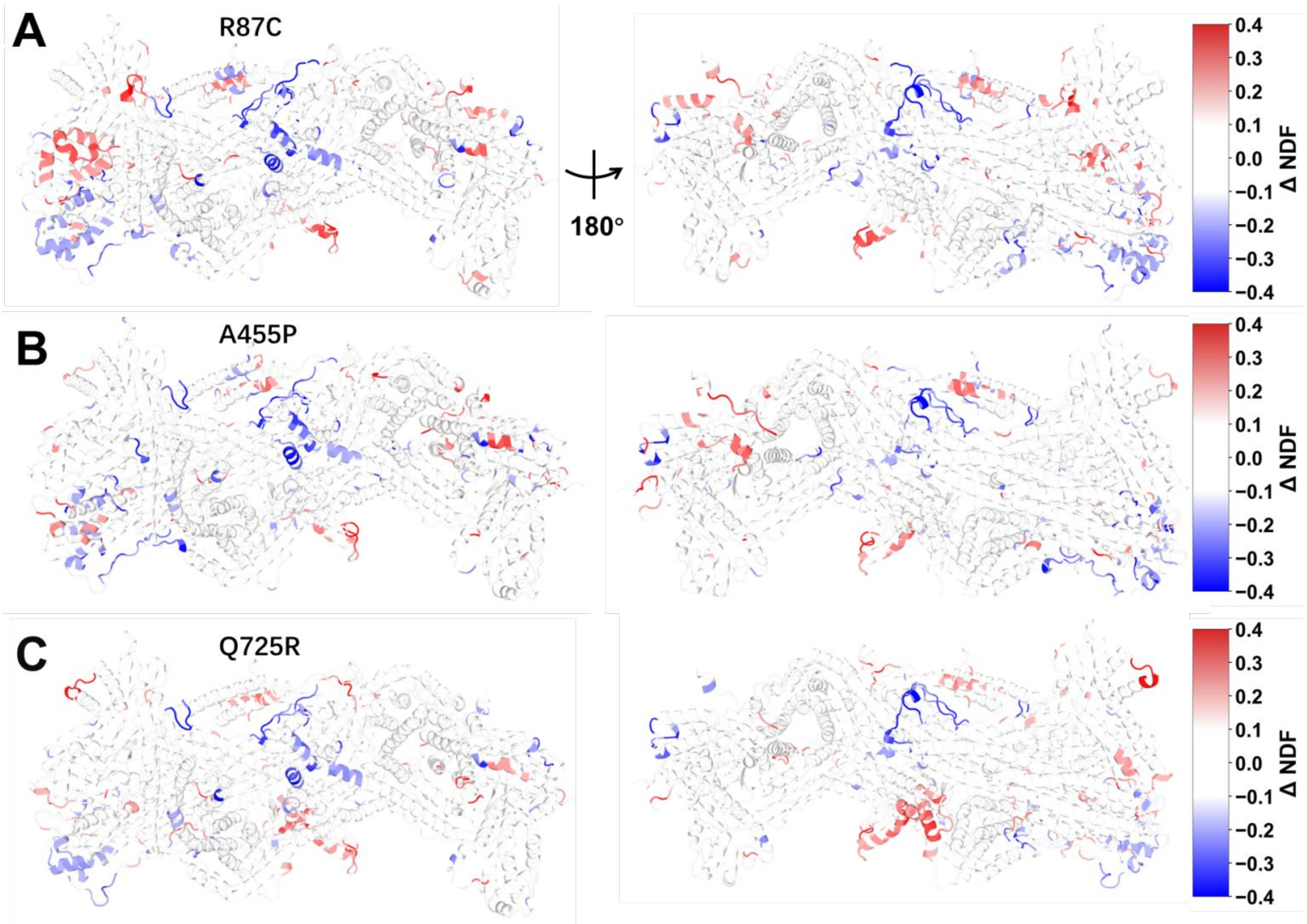

Figure S7. The ΔNDF_i values (i=1, 2, … 2924) for the four systems range from -0.4 (blue) to +0.4 (red). The proteins are shown as cartoons.

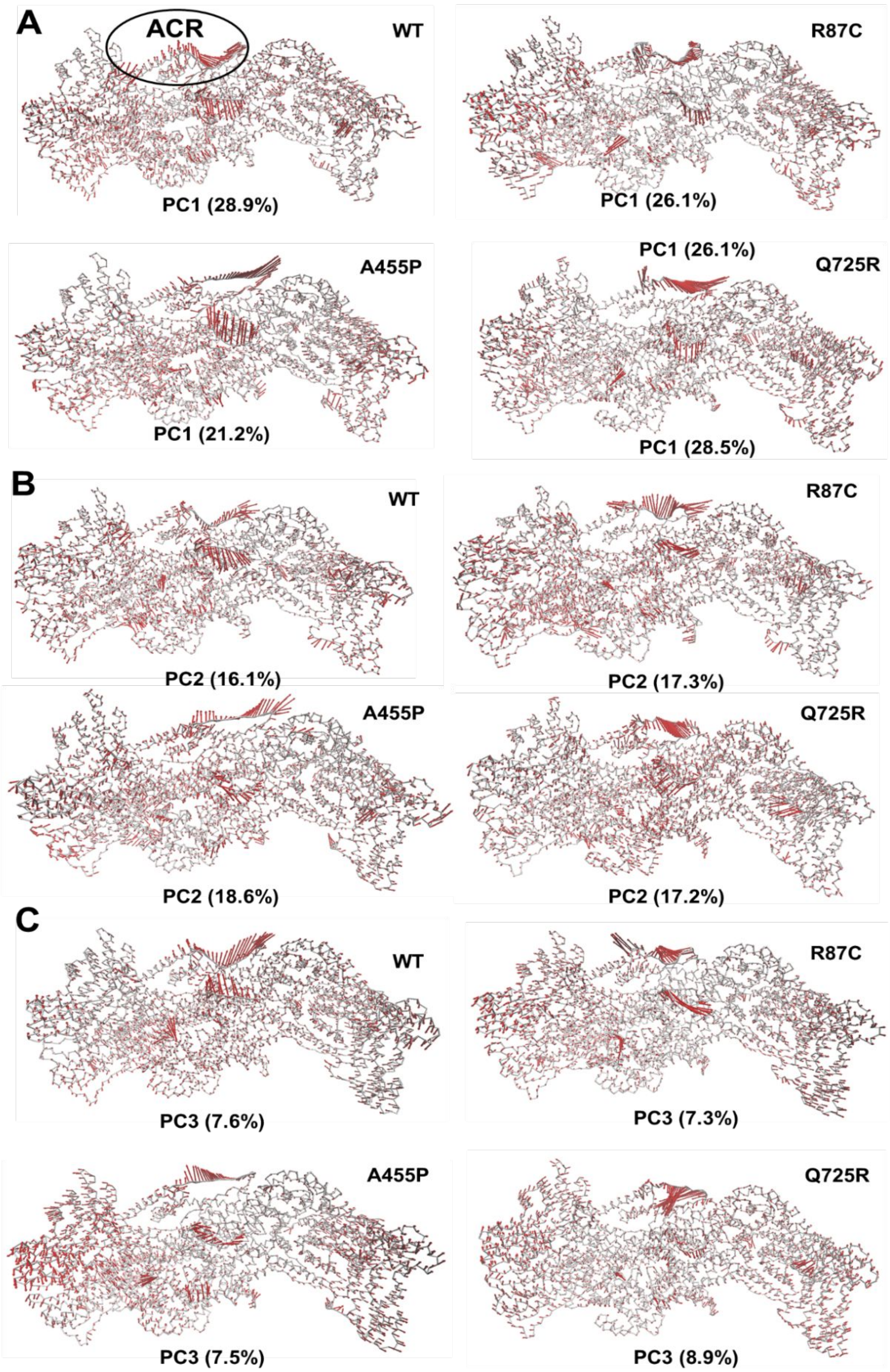

Figure S8. (A-C) The largest three eigenvectors (PC1-PC3) from our principal component analysis on the four systems. The percentage of total motion variance by each eigenvector is given in parentheses. The protein is displayed as a white ribbon; the direction of motion is shown as a red arrow.

Supporting Table

Table S1. Known NDDs-linked variants of CYFIP2.

Uniprot ID	Variant	Reference
**Q96F07-1 (iso1, canonical)**	p.Arg87Cys	[24]
	p.Arg87Leu	[24]
	p.Arg87Pro	[24]
	p.Tyr108His	[25]
	p.Ala455Pro	[25]
	p.Ile664Met	[25]
	p.Glu665Lys	[25]
	p.Asp724His	[25]
	p.Gln725Arg	[25]
**Q96F07-2 (iso2)**	p.Val14Met	[26]
	p.Arg87Cys	[26]
	p.Val181Phe	[26]
	p.Ala481Val	[27]
**E7EVJ5 (iso3, computationally mapped)**	p.Ile613Met	[26]
	p.Glu614Lys	[26]
	p.Tyr639Cys	[26]

	p.Gln674Arg	[26]
	p.Arg744Cys	[26]

Table S2. The pathogenic scores of three mutations predicted by PMUT[28] and MutPred2[29] servers.

Mutation	PMUT	MutPred2
R87C	0.85	0.85
A455P	0.56	0.91
I664M	0.85	0.63
E665K	0.78	0.81
D724H	0.87	0.77
Q725R	0.74	0.76

Table S3. Information about simulated systems.

System	Number of Na^+	Number of Cl^-	Number of water molecules
WT WRC	399	382	106,333
R87C WRC	397	379	106,018
A455P WRC	396	379	106,030
Q725R WRC	397	381	106,280

Table S4. The sequences used for building the WT model.

Protein (Uniprot ID)	Sequence
CYFIP2 (Q96F07-1)	MTTHVTLEDALSNVDLLEELPLPDQQPCIEPPPSSIMYQANFDTNFEDRNAFV<br>TGIARYIEQATVHSSMNEMLEEGHEYAVMLYTWRSCSRAIPQVKCNEQPNR<br>VEIYEKTVEVLEPEVTKLMKFMYFQRKAIERFCSEVKRLCHAERRKDFVSEA<br>YLLTLGKFINMFAVLDELKNMKCSVKNDHSAYKRAAQFLRKMADPQSIQES<br>QNLSMFLANHNRITQCLHQQLEVIPGYEELLADIVNICVDYYENKMYLTPSE<br>KHMLLKVMGFGLYLMDGNVSNIYKLDAKKRINLSKIDKFFKQLQVVPLFGD<br>MQIELARYIKTSAHYEENKSKWTCTQSSISPQYNICEQMVQIRDDHIRFISELA<br>RYSNSEVVTGSGLDSQKSDEEYRELFDLALRGLQLLSKWSAHVMEVYSWK<br>LVHPTDKFCNKDCPGTAEEYERATRYNYTSEEKFAFVEVIAMIKGLQVLMG

	RMESVFNQAIRNTIYAALQDFAQVTLREPLRQAVRKKKNVLISVLQAIRKTIC DWEGGREPPNDPCLRGEKDPKGGFDIKVPRRAVGPSSTQACQWSPRALFHP TGGTQGRRGCRSLLYMVRTMLESLIADKSGSKKTLRSSLDGPIVLAIEDFHK QSFFFTHLLNISEALQQCCDLSQLWFREFFLELTMGRRIQFPIEMSMPWILTD HILETKEPSMMEYVLYPLDLYNDSAYYALTKFKKQFLYDEIEAEVNLCFDQF VYKLADQIFAYYKAMAGSVLLDKRFRAECKNYGVIIPYPPSNRYETLLKQR HVQLLGRSIDLNRLITQRISAAMYKSLDQAISRFESEDLTSIVELEWLLEINRL THRLLCKHMTLDSFDAMFREANHNVSAPYGRITLHVFWELNFDFLPNYCYN GSTNRFVRTAIPFTQEPQRDKPANVQPYYLYGSKPLNIAYSHIYSSYRNFVGP PHFKTICRLLGYQGIAVVMEELLKIVKSLLQGTILQYVKTLIEVMPKICRLPR HEYGSPGILEFFHHQLKDIIEYAELKTDVFQSLREVGNAILFCLLIEQALSQEE VCDLLHAAPFQNILPRVYIKEGERLEVRMKRLEAKYAPLHLVPLIERLGTPQ QIAIAREGDLLTKERLCCGLSMFEVILTRIRSYLQDPIWRGPPPTNGVMHVDE CVEFHRLWSAMQFVYCIPVGTNEFTAEQCFGDGLNWAGCSIIVLLGQQRRF DLFDFCYHLLKVQRQDGKDEIIKNVPLKKMADRIRKYQILNNEVFAILNKYM KSVETDSSTVEHVRCFQPPIHQSLATTC
NCKAP1 (Q9Y2A7-2)	MSRSVLQPSQQKLAEKLTILNDRGVGMLTRLYNIKKQGQVWKACGDPKAK PSYLIDKNLESAVKFIVRKFPAVETRNNNQQLAQLQKEKSEILKNLALYYFTF VDVMEFKDHVCELLNTIDVCQVFFDITVNFDLTKNYLDLIITYTTLMILLSRIE ERKAIIGLYNYAHEMTHGASDREYPRLGQMIVDYENPLKKMMEEFVPHSKS LSDALISLQMVYPRRNLSADQWRNAQLLSLISAPSTMLNPAQSDTMPCEYLS LDAMEKWIIFGFILCHGILNTDATALNLWKLALQSSSCLSLFRDEVFHIHKAA EDLFVNIRGYNKRINDIRECKEAAVSHAGSMHRERRKFLRSALKELATVLSD QPGLLGPKALFVFMALSFARDEIIWLLRHADNMPKKSADDFIDKHIAELIFY MEELRAHVRKYGPVMQRYYVQYLSGFDAVVLNELVQNLSVCPEDESIIMSS FVNTMTSLSVKQVEDGEVFDFRGMRLDWFRLQAYTSVSKASLGLADHREL GKMMNTIIFHTKMVDSLVEMLVETSDLSIFCFYSRAFEKMFQQCLELPSQSR YSIAFPLLCTHFMSCTHELCPEERHHIGDRSLSLCNMFLDEMAKQARNLITDI CTEQCTLSDQLLPKHCAKTISQAVNKKSKKQTGKKGEPEREKPGVESMRKN RLVVTNLDKLHTALSELCFSINYVPNMVVWEHTFTPREYLTSHLEIRFTKSIV GMTMYNQATQEIAKPSELLTSVRAYMTVLQSIENYVQIDITRVFNNVLLQQT QHLDSHGEPTITSLYTNWYLETLLRQVSNGHIAYFPAMKAFVNLPTENELTF NAEEYSDISEMRSLSELLGPYGMKFLSESLMWHISSQVAELKKLVVENVDVL TQMRTSFDKPDQMAALFKRLSSVDSVLKRMTIIGVILSFRSLAQEALRDVLS YHIPFLVSSIEDFKDHIPRETDMKVAMNVYELSSAAGLPCEIDPALVVALSSQ KSENISPEEEYKIACLLMVFVAVSLPTLASNVMSQYSPAIEGHCNNIHCLAKA INQIAAALFTIHKGSIEDRLKEFLALASSSLLKIGQETDKTTTRNRESVYLLLD MIVQESPFLTMDLLESCFPYVLLRNAYHAVYKQSVTSSA
WAVE1 (Q92558)	MPLVKRNIDPRHLCHTALPRGIKNELECVTNISLANIIRQLSSLSKYAEDIFGE LFNEAHSFSFRVNSLQERVDRLSVSVTQLDPKEEELSLQDITMRKAFRSSTIQ DQQLFDRKTLPIPLQETYDVCEQPPPLNILTPYRDDGKEGLKFYTNPSYFFDL WKEKMLQDTEDKRKEKRKQKQKNLDRGGSGGSGGSGGSGGSGGSKRHPST LPVISDARSVLLEAIRKGIQLRKVEEQREQEAKHERIENDVATILSRRIAVEYS DSEDDSEFDEVDWLE
HSPC300 (Q8WUW1-1)	MAGQEDPVQREIHQDWANREYIEIITSSIKKIADFLNSFDMSCRSRLATLNEK LTALERRIEYIEARVTKGETLT

ABI2 (Q9NYB9-1)	MAELQMLLEEEIPGGRRALFDSYTNLERVADYCENNYIQSADKQRALEETK AYTTQSLASVAYLINTLANNVLQMLDIQASQLRRMESSINHISQTVDIHKEK VARREIGILTTNKNTSRTHKIIAPANLERPVRYIRKPIDYTILDDIGHGVKWLL R

Table S5. The Cα RMSD between our predicted WT WRC and available crystal structures of the complex.

PDB ID	Methodology	RMSD (Å)
3P8C [3]	X-ray	0.4
4N78[4]	X-ray	0.5
7USC[5]	Cryo-EM	0.9

Table S6. Occupancy of the H-bonds and contact scores formed by residues undergoing mutations in the four systems investigated here.

Residue pairs	WT	R87C	A455P	Q725R
	H-bond occupancy			
R87(CYFIP2)/Y151(ACR) or C87(CYFIP2)/Y151(ACR)	84%	2%	88%	92%
Q725(CYFIP2)/W161(ACR) or R725(CYFIP2)/W161(ACR)	46%	41%	56%	2%
	Contact scores			
R87(CYFIP2)/Y151(ACR) or C87(CYFIP2)/Y151(ACR)	41	27	39	39
A455(CYFIP2)/Y687(ACR) or P455(CYFIP2)/Y687(ACR)	7	6	15	7
A455(CYFIP2)/Y690(ACR) or P455(CYFIP2)/Y690(ACR)	21	24	22	22
Q725(CYFIP2)/V531(ACR) or R725(CYFIP2)/V531(ACR)	5	6	5	5
Q725(CYFIP2)/L535(ACR) or R725(CYFIP2)/L535(ACR)	5	4	4	1

Table S7. Cosine similarity[30]among the difference motion correlation matrices on passing from variants to the WT.

	**R87C**	**A455P**	**Q725R**
**R87C**	1	0.54	0.56
**A455P**	0.54	1	0.48
**Q725R**	0.56	0.48	1

Table S8. Spearman correlation[31] among the difference motion correlation matrices on passing from variants to the WT.

	**R87C**	**A455P**	**Q725R**
**R87C**	1	0.5	0.53
**A455P**	0.5	1	0.44
**Q725R**	0.53	0.44	1

Table S9. Known mutations affecting structure and/or affinity among PPIs in ASD.

Protein	Mutation	Effect	Damage	Reference
SH3 and multiple ankyrin repeat domains 3 (Shank3)	L68P	Increase the binding of Shank3 to cytoskeletal protein α-fodrin	Actin cytoskeleton of the dendritic spin and postsynaptic signal transduction	32
Calcium/calmodulin-dependent protein kinase II (CaMKII)	E183V	Decrease the binding of CaMKII to Shank3 and subunits of l-type calcium channels and N-methyl-D-aspartic acid (NMDA) receptors.	Synaptic functions	33
Secreted protein acidic and rich in cysteine-like 1 (Sparcl1)	W647R	Increase the binding of Sparcl1 to binding immunoglobulin protein (BIP)	Synaptic function	34
Eukaryotic Elongation Factor 1A2 (eEF1A2)	E122K and D252H	E122K and D252H increase the binding of eEF1A2 to tRNA. D252H disrupts the binding of eEF1A2 to eEF1 complex (composed of composed of eEF1B2, eEF1D, and eEF1G)	Actin cytoskeleton in neuron	35
ArfGAP with dual PH domain-containing protein 1 (ADAP1)	G144R	Decrease the binding of ADAP1 to kinesin Family Member 13B (KIF13B)	Neuronal polarity formation and axon specification	36
Retinoic Acid Receptor Alpha (RARA)	P375L	Decrease the binding of RARA to Retinoid X Receptor Beta (RXRB)	Neuronal development and synaptic plasticity	37
Fragile X mental retardation protein (FMRP)	I304N	Decrease the binding of FMRP to RNA, HDAC1, DYRK1A, CUL3, CHD8, and POGZ	Dendritic morphogenesis and altered axonal targeting,	38, 39

Table S10. Largest changes in contact scores of the residue pairs (see Method section) at the interface between ACR and the rest of WRC, on passing from the variants to the WT. Negative changes denote weaker interactions in the variants relative to WT. Positive changes are the other way around.

	The difference in contact scores (Δ variants, WT)

R87C	
E718(CYFIP2)/F157(ACR)	-15
R87(CYFIP2)/Y151(ACR) or C87(CYFIP2)/Y151(ACR)	-14
Y972(CYFIP2)/L491(ACR)	-11
P909(CYFIP2)/Y543(ACR)	11
N906(CYFIP2)/A540(ACR)	10
A455P	
Q661(CYFIP2)/R156(ACR)	13
Q897(CYFIP2)/K485(ACR)	-8
Y910(CYFIP2)/Y543(ACR)	-8
R103(WAVE1)/E516(ACR)	-8
D1016(CYFIP2)/S489(ACR)	-7
Q725R	
Q897(CYFIP2)/R486(ACR)	-9
Y910(CYFIP2)/Y543(ACR)	-8
Y728(CYFIP2)/E559(ACR)	-8
D1016(CYFIP2)/S489(ACR)	-7
R795(CYFIP2)/E559(ACR)	-7

Supporting references

1. Xie, S.; Zuo, K.; De Rubeis, S.; Ruggerone, P.; Carloni, P., Molecular basis of the CYFIP2 and NCKAP1 autism-linked variants in the WAVE regulatory complex. *Protein Science* **2025**, 34, e5238.
2. Abramson, J.; Adler, J.; Dunger, J.; Evans, R.; Green, T.; Pritzel, A.; Ronneberger, O.; Willmore, L.; Ballard, A. J.; Bambrick, J.; Bodenstein, S. W.; Evans, D. A.; Hung, C.-C.; O'Neill, M.; Reiman, D.; Tunyasuvunakool, K.; Wu, Z.; Žemgulytė, A.; Arvaniti, E.; Beattie, C.; Bertolli, O.; Bridgland, A.; Cherepanov, A.; Congreve, M.; Cowen-Rivers, A. I.; Cowie, A.; Figurnov, M.; Fuchs, F. B.; Gladman, H.; Jain, R.; Khan, Y. A.; Low, C. M. R.; Perlin, K.; Potapenko, A.; Savy, P.; Singh, S.; Stecula, A.; Thillaisundaram, A.; Tong, C.; Yakneen, S.; Zhong, E. D.; Zielinski, M.; Žídek, A.; Bapst, V.; Kohli, P.; Jaderberg, M.; Hassabis, D.; Jumper, J. M., Accurate structure prediction of biomolecular interactions with AlphaFold 3. *Nature* **2024**, 630, 493-500.
3. Chen, Z.; Borek, D.; Padrick, S. B.; Gomez, T. S.; Metlagel, Z.; Ismail, A. M.; Umetani, J.; Billadeau, D. D.; Otwinowski, Z.; Rosen, M. K., Structure and control of the actin regulatory WAVE complex. *Nature* **2010**, 468, 533-538.
4. Chen, B.; Brinkmann, K.; Chen, Z.; Pak, Chi W.; Liao, Y.; Shi, S.; Henry, L.; Grishin, Nick V.; Bogdan, S.; Rosen, Michael K., The WAVE Regulatory Complex Links Diverse Receptors to the Actin Cytoskeleton. *Cell* **2014**, 156, 195-207.
5. Ding, B.; Yang, S.; Schaks, M.; Liu, Y.; Brown, A. J.; Rottner, K.; Chowdhury, S.; Chen, B., Structures reveal a key mechanism of WAVE regulatory complex activation by Rac1 GTPase. *Nature Communications* **2022**, 13, 5444.
6. Waterhouse, A.; Bertoni, M.; Bienert, S.; Studer, G.; Tauriello, G.; Gumienny, R.; Heer, F. T.; de Beer, T. A P.; Rempfer, C.; Bordoli, L.; Lepore, R.; Schwede, T., SWISS-MODEL: homology modelling of protein structures and complexes. *Nucleic Acids Research* **2018**, 46, W296-W303.
7. Gordon, J. C.; Myers, J. B.; Folta, T.; Shoja, V.; Heath, L. S.; Onufriev, A., H++: a server for estimating p Ka s and adding missing hydrogens to macromolecules. *Nucleic Acids Research* **2005**, 33, W368-W371.
8. Case, D. A.; Cheatham III, T. E.; Darden, T.; Gohlke, H.; Luo, R.; Merz Jr., K. M.; Onufriev, A.; Simmerling, C.; Wang, B.; Woods, R. J., The Amber biomolecular simulation programs. *Journal of Computational Chemistry* **2005**, 26, 1668-1688.
9. Tian, C.; Kasavajhala, K.; Belfon, K. A. A.; Raguette, L.; Huang, H.; Migues, A. N.; Bickel, J.; Wang, Y.; Pincay, J.; Wu, Q.; Simmerling, C., ff19SB: Amino-Acid-Specific Protein Backbone Parameters Trained against Quantum Mechanics Energy Surfaces in Solution. *Journal of Chemical Theory and Computation* **2020**, 16, 528-552.
10. Izadi, S.; Anandakrishnan, R.; Onufriev, A. V., Building Water Models: A Different Approach. *The Journal of Physical Chemistry Letters* **2014**, 5, 3863-3871.
11. Åqvist, J., Ion-water interaction potentials derived from free energy perturbation simulations. *The Journal of Physical Chemistry* **1990**, 94, 8021-8024.
12. Darden, T.; York, D.; Pedersen, L., Particle mesh Ewald: An N·log(N) method for Ewald sums in large systems. *The Journal of Chemical Physics* **1993**, 98, 10089-10092.
13. Lemons, D. S.; Gythiel, A., Paul Langevin's 1908 paper "On the Theory of Brownian Motion" ["Sur la théorie du mouvement brownien," C. R. Acad. Sci. (Paris) 146, 530–533 (1908)]. *American Journal of Physics* **1997**, 65, 1079-1081.
14. Allen, M. P.; Tildesley, D. J., *Computer Simulation of Liquids*. Oxford University Press: 2017.
15. Ester, M.; Kriegel, H.-P.; Sander, J.; Xu, X., In *Proceedings of the Second International Conference on Knowledge Discovery and Data Mining*; AAAI Press: Portland, Oregon, 1996, pp 226–231.
16. Weiser, J.; Shenkin, P. S.; Still, W. C., Approximate atomic surfaces from linear combinations of pairwise overlaps (LCPO). *Journal of Computational Chemistry* **1999**, 20, 217-230.
17. Ichiye, T.; Karplus, M., Collective motions in proteins: A covariance analysis of atomic fluctuations in molecular dynamics and normal mode simulations. *Proteins: Structure, Function, and Bioinformatics* **1991**, 11, 205-217.
18. García, A. E., Large-amplitude nonlinear motions in proteins. *Physical Review Letters* **1992**, 68, 2696-2699.

19. Morra, G.; Potestio, R.; Micheletti, C.; Colombo, G., Corresponding Functional Dynamics across the Hsp90 Chaperone Family: Insights from a Multiscale Analysis of MD Simulations. *PLOS Computational Biology* **2012**, 8, e1002433.
20. Exploratory Data Analysis. In *The Concise Encyclopedia of Statistics*; Springer New York: New York, NY, 2008, pp 192-194.
21. Malhotra, P.; Udgaonkar, J. B., How cooperative are protein folding and unfolding transitions? *Protein Science* **2016**, 25, 1924-1941.
22. Laursen, L.; Gianni, S.; Jemth, P., Dissecting Inter-domain Cooperativity in the Folding of a Multi Domain Protein. *Journal of Molecular Biology* **2021**, 433, 167148.
23. Roe, D. R.; Cheatham, T. E., III, PTRAJ and CPPTRAJ: Software for Processing and Analysis of Molecular Dynamics Trajectory Data. *Journal of Chemical Theory and Computation* **2013**, 9, 3084-3095.
24. Nakashima, M.; Kato, M.; Aoto, K.; Shiina, M.; Belal, H.; Mukaida, S.; Kumada, S.; Sato, A.; Zerem, A.; Lerman-Sagie, T.; Lev, D.; Leong, H. Y.; Tsurusaki, Y.; Mizuguchi, T.; Miyatake, S.; Miyake, N.; Ogata, K.; Saitsu, H.; Matsumoto, N., De novo hotspot variants in CYFIP2 cause early-onset epileptic encephalopathy. *Annals of Neurology* **2018**, 83, 794-806.
25. Zweier, M.; Begemann, A.; McWalter, K.; Cho, M. T.; Abela, L.; Banka, S.; Behring, B.; Berger, A.; Brown, C. W.; Carneiro, M.; Chen, J.; Cooper, G. M.; Finnila, C. R.; Guillen Sacoto, M. J.; Henderson, A.; Hüffmeier, U.; Joset, P.; Kerr, B.; Lesca, G.; Leszinski, G. S.; McDermott, J. H.; Meltzer, M. R.; Monaghan, K. G.; Mostafavi, R.; Õunap, K.; Plecko, B.; Powis, Z.; Purcarin, G.; Reimand, T.; Riedhammer, K. M.; Schreiber, J. M.; Sirsi, D.; Wierenga, K. J.; Wojcik, M. H.; Papuc, S. M.; Steindl, K.; Sticht, H.; Rauch, A.; Deciphering Developmental Disorders, S., Spatially clustering de novo variants in CYFIP2, encoding the cytoplasmic FMRP interacting protein 2, cause intellectual disability and seizures. *European Journal of Human Genetics* **2019**, 27, 747-759.
26. Firth, H. V.; Richards, S. M.; Bevan, A. P.; Clayton, S.; Corpas, M.; Rajan, D.; Vooren, S. V.; Moreau, Y.; Pettett, R. M.; Carter, N. P., DECIPHER: *Database of Chromosomal Imbalance and Phenotype in Humans Using Ensembl Resources. The American Journal of Human Genetics* **2009**, 84, 524-533.
27. Fu, J. M.; Satterstrom, F. K.; Peng, M.; Brand, H.; Collins, R. L.; Dong, S.; Wamsley, B.; Klei, L.; Wang, L.; Hao, S. P.; Stevens, C. R.; Cusick, C.; Babadi, M.; Banks, E.; Collins, B.; Dodge, S.; Gabriel, S. B.; Gauthier, L.; Lee, S. K.; Liang, L.; Ljungdahl, A.; Mahjani, B.; Sloofman, L.; Smirnov, A. N.; Barbosa, M.; Betancur, C.; Brusco, A.; Chung, B. H. Y.; Cook, E. H.; Cuccaro, M. L.; Domenici, E.; Ferrero, G. B.; Gargus, J. J.; Herman, G. E.; Hertz-Picciotto, I.; Maciel, P.; Manoach, D. S.; Passos-Bueno, M. R.; Persico, A. M.; Renieri, A.; Sutcliffe, J. S.; Tassone, F.; Trabetti, E.; Campos, G.; Cardaropoli, S.; Carli, D.; Chan, M. C. Y.; Fallerini, C.; Giorgio, E.; Girardi, A. C.; Hansen-Kiss, E.; Lee, S. L.; Lintas, C.; Ludena, Y.; Nguyen, R.; Pavinato, L.; Pericak-Vance, M.; Pessah, I. N.; Schmidt, R. J.; Smith, M.; Costa, C. I. S.; Trajkova, S.; Wang, J. Y. T.; Yu, M. H. C.; Aleksic, B.; Artomov, M.; Benetti, E.; Biscaldi-Schafer, M.; Børglum, A. D.; Carracedo, A.; Chiocchetti, A. G.; Coon, H.; Doan, R. N.; Fernández-Prieto, M.; Freitag, C. M.; Gerges, S.; Guter, S.; Hougaard, D. M.; Hultman, C. M.; Jacob, S.; Kaartinen, M.; Kolevzon, A.; Kushima, I.; Lehtimäki, T.; Rizzo, C. L.; Maltman, N.; Manara, M.; Meiri, G.; Menashe, I.; Miller, J.; Minshew, N.; Mosconi, M.; Ozaki, N.; Palotie, A.; Parellada, M.; Puura, K.; Reichenberg, A.; Sandin, S.; Scherer, S. W.; Schlitt, S.; Schmitt, L.; Schneider-Momm, K.; Siper, P. M.; Suren, P.; Sweeney, J. A.; Teufel, K.; del Pilar Trelles, M.; Weiss, L. A.; Yuen, R.; Cutler, D. J.; De Rubeis, S.; Buxbaum, J. D.; Daly, M. J.; Devlin, B.; Roeder, K.; Sanders, S. J.; Talkowski, M. E.; The Autism Sequencing, C.; Broad Institute Center for Common Disease, G.; i, P.-B. C., Rare coding variation provides insight into the genetic architecture and phenotypic context of autism. *Nature Genetics* **2022**, 54, 1320-1331.
28. López-Ferrando, V.; Gazzo, A.; de la Cruz, X.; Orozco, M.; Gelpí, J. L., PMut: a web-based tool for the annotation of pathological variants on proteins, 2017 update. *Nucleic Acids Research* **2017**, 45, W222-W228.
29. Pejaver, V.; Urresti, J.; Lugo-Martinez, J.; Pagel, K. A.; Lin, G. N.; Nam, H.-J.; Mort, M.; Cooper, D. N.; Sebat, J.; Iakoucheva, L. M.; Mooney, S. D.; Radivojac, P., Inferring the molecular and phenotypic impact of amino acid variants with MutPred2. *Nature Communications* **2020**, 11, 5918.
30. Salton, G.; Wong, A.; Yang, C. S., A vector space model for automatic indexing. *Commun. ACM* **1975**, 18, 613–620.
31. Sedgwick, P., Spearman's rank correlation coefficient. *BMJ* **2014**, 349, g7327.
32. Mameza, M. G.; Dvoretskova, E.; Bamann, M.; Hönck, H.-H.; Güler, T.; Boeckers, T. M.; Schoen, M.; Verpelli, C.; Sala, C.; Barsukov, I.; Dityatev, A.; Kreienkamp, H.-J., SHANK3 Gene Mutations

Associated with Autism Facilitate Ligand Binding to the Shank3 Ankyrin Repeat Region*. *Journal of Biological Chemistry* **2013**, 288, 26697-26708.
33. Stephenson, J. R.; Wang, X.; Perfitt, T. L.; Parrish, W. P.; Shonesy, B. C.; Marks, C. R.; Mortlock, D. P.; Nakagawa, T.; Sutcliffe, J. S.; Colbran, R. J., A Novel Human *CAMK2A* Mutation Disrupts Dendritic Morphology and Synaptic Transmission, and Causes ASD-Related Behaviors. *The Journal of Neuroscience* **2017**, 37, 2216-2233.
34. Taketomi, T.; Yasuda, T.; Morita, R.; Kim, J.; Shigeta, Y.; Eroglu, C.; Harada, R.; Tsuruta, F., Autism-associated mutation in Hevin/Sparcl1 induces endoplasmic reticulum stress through structural instability. *Scientific Reports* **2022**, 12, 11891.
35. Mohamed, M. S.; Klann, E., Autism- and epilepsy-associated EEF1A2 mutations lead to translational dysfunction and altered actin bundling. *Proceedings of the National Academy of Sciences* **2023**, 120, e2307704120.
36. Chen, S.; Wang, J.; Cicek, E.; Roeder, K.; Yu, H.; Devlin, B., De novo missense variants disrupting protein–protein interactions affect risk for autism through gene co-expression and protein networks in neuronal cell types. *Molecular Autism* **2020**, 11, 76.
37. Chen, S.; Fragoza, R.; Klei, L.; Liu, Y.; Wang, J.; Roeder, K.; Devlin, B.; Yu, H., An interactome perturbation framework prioritizes damaging missense mutations for developmental disorders. *Nature Genetics* **2018**, 50, 1032-1040.
38. Ascano, M.; Mukherjee, N.; Bandaru, P.; Miller, J. B.; Nusbaum, J. D.; Corcoran, D. L.; Langlois, C.; Munschauer, M.; Dewell, S.; Hafner, M.; Williams, Z.; Ohler, U.; Tuschl, T., FMRP targets distinct mRNA sequence elements to regulate protein expression. *Nature* **2012**, 492, 382-386.
39. Li, J.; Ma, Z.; Shi, M.; Malty, Ramy H.; Aoki, H.; Minic, Z.; Phanse, S.; Jin, K.; Wall, Dennis P.; Zhang, Z.; Urban, Alexander E.; Hallmayer, J.; Babu, M.; Snyder, M., Identification of Human Neuronal Protein Complexes Reveals Biochemical Activities and Convergent Mechanisms of Action in Autism Spectrum Disorders. *Cell Systems* **2015**, 1, 361-374.
